# FBENet: A Highway Road Debris Detection Network Based on Frequency-Aware Bidirectional Feature Fusion and Efficient Attention Enhancement

**DOI:** 10.3390/s26165062

**Published:** 2026-08-10

**Authors:** Yange Chen, Baohua Guo, Sen Wang, Anthony Sigama, David Bassir

**Affiliations:** 1 School of Energy Science and Engineering, Henan Polytechnic University, Jiaozuo 454003, China; 18438045760@163.com (Y.C.); wangsen7719@163.com (S.W.); anthonymsigama@gmail.com (A.S.); 2 Jiaozuo Engineering Research Center of Road Traffic and Transportation, Henan Polytechnic University, Jiaozuo 454000, China; 3 Faculty of Science, Engineering and Agriculture, University of Venda, Thohoyandou 0950, South Africa; 4 Smart Structural Health Monitoring and Control Laboratory, DGUT-CNAM Institute, Dongguan University of Technology, Dongguan 523808, China; 5 Centre Borelli, UMR CNRS 9010, Saclay Campus, École Normale Supérieure Paris-Saclay, Component Institution of Université Paris-Saclay, 4, Avenue des Sciences, 91190 Gif-sur-Yvette, France

**Keywords:** highway road debris, synthetic datasets, object detection, frequency-aware, efficient multi-scale attention, YOLO11n

## Abstract

**Highlights:**

**What are the main findings?**

- FBENet (Frequency-Aware Bidirectional Feature Fusion and Efficient Attention Enhancement Network) achieved an mAP@0.5 of 0.862 and an F1-score of 0.811 on Synthetic-Debris.
- FBENet (Frequency-Aware Bidirectional Feature Fusion and Efficient Attention Enhancement Network) showed modest overall synthetic-to-real transfer gains over YOLO11n, with class-dependent performance.

**What are the implications of the main findings?**

- Frequency-aware fusion and selective EMA attention strengthen small-debris representation.
- Synthetic training can support transfer when real debris samples are limited.

**Abstract:**

Highway road debris is often small, irregular, weakly textured, and poorly contrasted against complex backgrounds, while limited real-world samples constrain model generalization. This study constructs a Synthetic-Debris dataset by compositing three representative debris categories—brick, paper box, and rock—onto highway scenes to support small-object and low-contrast detection. FBENet (Frequency-Aware Bidirectional Feature Fusion and Efficient Attention Enhancement Network) is developed from YOLO11n using a task-oriented, stage-coupled design: FreqFusion (Frequency-aware Feature Fusion) is embedded at two top-down cross-scale fusion stages to preserve boundary details and improve cross-resolution consistency before learnable bidirectional aggregation by BiFPN (Bi-directional Feature Pyramid Network), while EMA (Efficient Multi-Scale Attention) is applied only to the final high-resolution P3 feature before detection. The contribution lies in the stage-specific organization of established operations rather than in proposing new primitive modules. On Synthetic-Debris, FBENet achieved an mAP@0.5 of 0.862, an mAP@0.5:0.95 of 0.664, and an F1-score of 0.811, with 2.43 M parameters and 6.8 GFLOPs. In the fixed synthetic-to-real split, FBENet exceeded YOLO11n by 5.0 and 2.8 percentage points in mAP@0.5 and mAP@0.5:0.95, respectively. Five-fold cross-validation showed modest mean AP gains and lower overall AP variance, although transfer behavior remained class-dependent. Runtime evaluation showed that FBENet incurred additional latency relative to YOLO11n. Under PyTorch FP32, the network inference latency increased from 8.07 to 12.79 ms, while the end-to-end throughput decreased from 79.15 to 53.97 FPS. Under TensorRT FP16, FBENet achieved 126.59 FPS compared with 137.64 FPS for YOLO11n. These results indicate that FBENet improves detection accuracy at the cost of additional runtime, representing an accuracy–latency trade-off rather than an improvement in inference efficiency. Overall, frequency-aware bidirectional fusion and selective high-resolution attention are useful for improving highway debris detection under limited real-data conditions.

## 1. Introduction

With the rapid development of intelligent transportation systems and automated driving, reliable road-environment perception has become essential for safe decision-making. Highway scenarios are particularly demanding because vehicles travel rapidly, traffic is dense, and braking distances are long. When highway road debris such as rock, paper box, or brick appears in a traffic lane, it may trigger emergency braking, abrupt evasive maneuvers, vehicle instability, or secondary collisions. For automated vehicles, delayed or missed detection of these infrequent but hazardous objects can impair obstacle avoidance and trajectory planning. Debris higher than 15 cm may cause rollover or trajectory deviation after impact [1]. Accurate, real-time detection of highway road debris is therefore important for autonomous driving and highway safety.

Compared with vehicles, pedestrians, and traffic signs, highway road debris is difficult to detect because of its small size, irregular shape, appearance variation, weak texture, and low contrast [2]. Under long-distance imaging, objects occupy few pixels and provide incomplete edge and contour information, making them easily confused with shadows, cracks, stains, lane wear, and heterogeneous pavement. High-speed driving also limits response time, requiring models to balance accuracy, robustness, efficiency, and deployment feasibility. Moreover, debris events are sporadic and hazardous to collect. Real samples are therefore limited in scale, class balance, viewpoint, illumination, weather, occlusion, and background diversity, increasing overfitting and weakening generalization.

Early detection methods primarily employed techniques such as background modeling [3], foreground extraction [4], inter-frame differencing [5,6], and static-object analysis. They can perform adequately with fixed cameras and stable scenes, but are sensitive to illumination changes, moving shadows, camera vibration, and complex pavement patterns. Deep learning has become dominant because it learns discriminative features directly from data. Multi-scale fusion, attention, lightweight convolution, and Transformer-based modeling improve small-object recognition and contextual reasoning [7]. The YOLO family [8,9,10] is attractive for traffic applications because of its end-to-end formulation, fast inference, and convenient deployment. However, standard YOLO detectors may provide insufficient cross-scale interaction, weak low-texture representation, and dispersed responses in cluttered backgrounds, causing missed detections, classification errors, and duplicate predictions.

Recent studies have developed specialized models for road debris and abandoned objects. Qian et al. combined a residual network, Selective Search, and non-maximum suppression to identify vehicle-thrown waste in surveillance videos [11]. Liu et al. used a lightweight backbone, depthwise separable convolution, attention, and knowledge distillation to improve freeway debris detection [12]. CIEFRNet strengthened small-target features through contextual modeling, spatial pyramid pooling, and attention [2], while an attention-based bidirectional feature pyramid improved real-time highway litter detection [13]. Multi-scale fusion and dynamic enhancement were applied to irregular objects [14], and lightweight Transformers captured global context [15]. YOLO with background differencing also demonstrated practical real-time performance [16]. Nevertheless, limited real samples and inadequate feature aggregation remain critical constraints for small, weak-texture targets. More broadly, Zunair et al. [17] introduced RSUD20K, a large-scale road-scene dataset containing over 20,000 high-resolution driving-view images with ~130,000 annotations across 13 categories. Capturing diverse conditions—narrow streets, highways, crowding, varied viewpoints, and weather—it has served as a benchmark for detectors and large vision model-based annotation.

Synthetic data can reduce sample scarcity and annotation cost. Simulation studies show that target scale, perspective, background composition, and environmental parameters strongly influence synthetic-to-real transfer [18]. Fourie et al. evaluated synthetic pretraining, real-data fine-tuning, and mixed training for YOLO-based crocodile detection [19]. Kim et al. inserted scaffold objects into construction scenes and examined different synthetic-to-real proportions [20], while Venkataramanan et al. generated seamlessly blended microscopy images and improved detection through synthetic pretraining and real-data fine-tuning [21]. Generative models and image fusion further increase diversity. Chen et al. combined language-assisted generation, diffusion models, and compositing to construct railway foreign-object images [22]. HazardNet embedded synthetic obstacles into real roads using semantic enhancement and domain randomization [1]. However, differences in illumination, texture, boundaries, imaging noise, and scene realism may cause models trained only on synthetic data to learn domain-specific artifacts instead of transferable debris features.

The main unresolved issue is not the absence of individual enhancement strategies, but the lack of a coordinated study linking data scarcity, feature representation, and real-scene transfer. Existing methods generally examine these aspects in isolation; few investigate how frequency-aware detail preservation, bidirectional cross-scale fusion, and selective spatial attention can be combined for small, low-contrast debris. In addition, synthetic-data studies are often evaluated primarily on generated images, leaving transfer to real highway scenes insufficiently characterized. To address these limitations, this study constructs a Synthetic-Debris dataset by compositing brick, paper box, and rock instances with diverse highway backgrounds and develops FBENet on the YOLO11n framework. FreqFusion is introduced at the two top-down fusion stages to improve boundary information and cross-resolution consistency; BiFPN performs weighted bidirectional aggregation, and EMA is applied only to the final P3 feature to refine spatial responses to small targets. The detector is first evaluated on the synthetic dataset and then assessed for synthetic-to-real transfer using a fixed split and five-fold cross-validation on real highway images. Runtime is also measured on the evaluated GPU platform to characterize the accuracy-efficiency trade-off.

The main contributions of this study are summarized as follows.

1. To alleviate the scarcity of real samples and the high cost of labeling in highway debris detection, a Synthetic-Debris dataset is constructed. Highway road images are used as background scenes, and three typical low-frequency yet high-risk debris categories, rock, paper box, and brick, are selected as foreground targets. Through compositing, data augmentation, and manual screening, diverse synthetic samples are generated to support training for small and low-contrast debris detection.
2. A task-oriented, stage-coupled feature-fusion architecture is developed for small and low-contrast highway debris. In the YOLO11n neck, FreqFusion is embedded at two top-down cross-scale fusion stages to improve high-frequency boundary details and cross-resolution consistency before BiFPN performs learnable bidirectional aggregation. EMA is then selectively applied to the final high-resolution P3 feature before detection.
3. To evaluate synthetic-to-real transfer under limited real data, experiments are conducted using both a fixed real-scene split and five-fold cross-validation. The evaluation reports class-wise AP@0.5, AP@0.5:0.95, precision, recall, and cross-fold variance. The results show modest overall improvements and lower overall AP variance compared with YOLO11n, while also revealing class-dependent transfer behavior.

## 2. Improved Model Design

### 2.1. Model Architecture

The YOLO11 [23] model has five models: n, s, m, l, and x. The tasks, parameter counts, and computation counts for each model are shown in Table 1.

Considering detection accuracy, inference efficiency, and model complexity, YOLO11n was selected as the baseline model for subsequent improvements.

**Figure 1:**
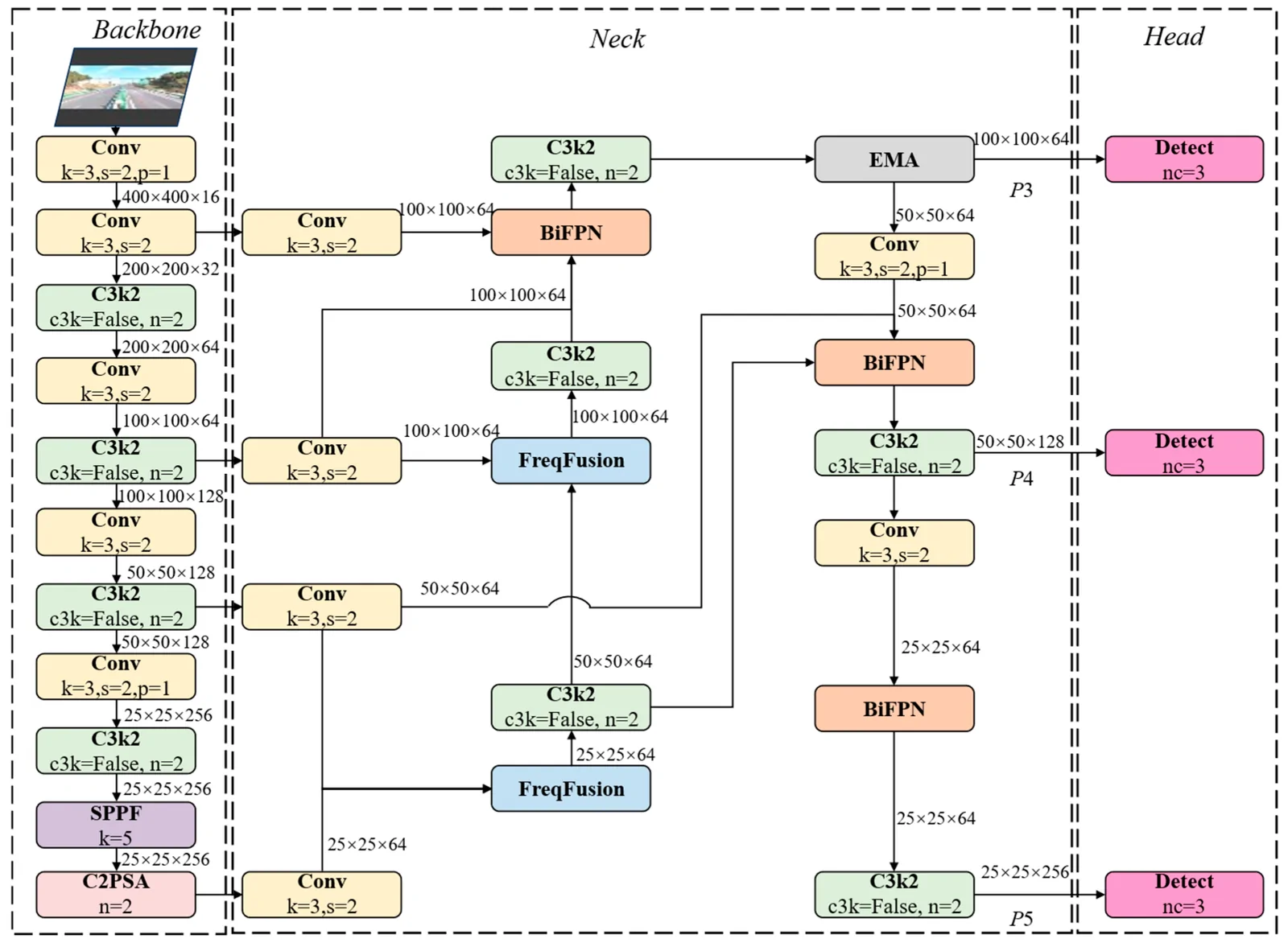
Overall architecture of FBENet. FreqFusion is introduced at the two top-down cross-scale fusion stages, BiFPN performs weighted bidirectional multi-scale aggregation, and EMA is selectively applied to the high-resolution P3 feature before detection. P3, P4, and P5 denote the three detection scales. Arrows denote the direction of feature flow.

Highway road debris is characterized by small scales, low contrast, weak edges, and strong interference from background textures. Relying solely on spatial-domain feature fusion can weaken object boundaries. To address these issues, we designed FBENet (Frequency-Aware Bidirectional Feature Fusion and Efficient Attention Enhancement Network). The design principle of FBENet is to assign different feature-enhancement mechanisms to the stages where they are most relevant to highway debris detection. FreqFusion is used at the top-down cross-scale fusion nodes to recover weak boundary details and reduce feature inconsistency during semantic upsampling. BiFPN subsequently performs learnable bidirectional aggregation of the frequency-enhanced features. After completion of the neck fusion process, EMA is inserted only before the P3 detection head to refine the final high-resolution representation. Accordingly, FBENet forms a sequential feature-processing pathway consisting of frequency-aware cross-scale enhancement, weighted bidirectional aggregation, and selective high-resolution spatial refinement. The architecture of FBENet is shown in Figure 1.

FBENet tackles critical issues such as edge detail degradation, inadequate cross-scale fusion, and lack of spatial attention through the co-design of an enhanced FreqFusion-BiFPN module and EMA, effectively boosting highway debris detection performance.

### 2.2. FreqFusion-BiFPN Feature-Fusion Neck

**Figure 2:**
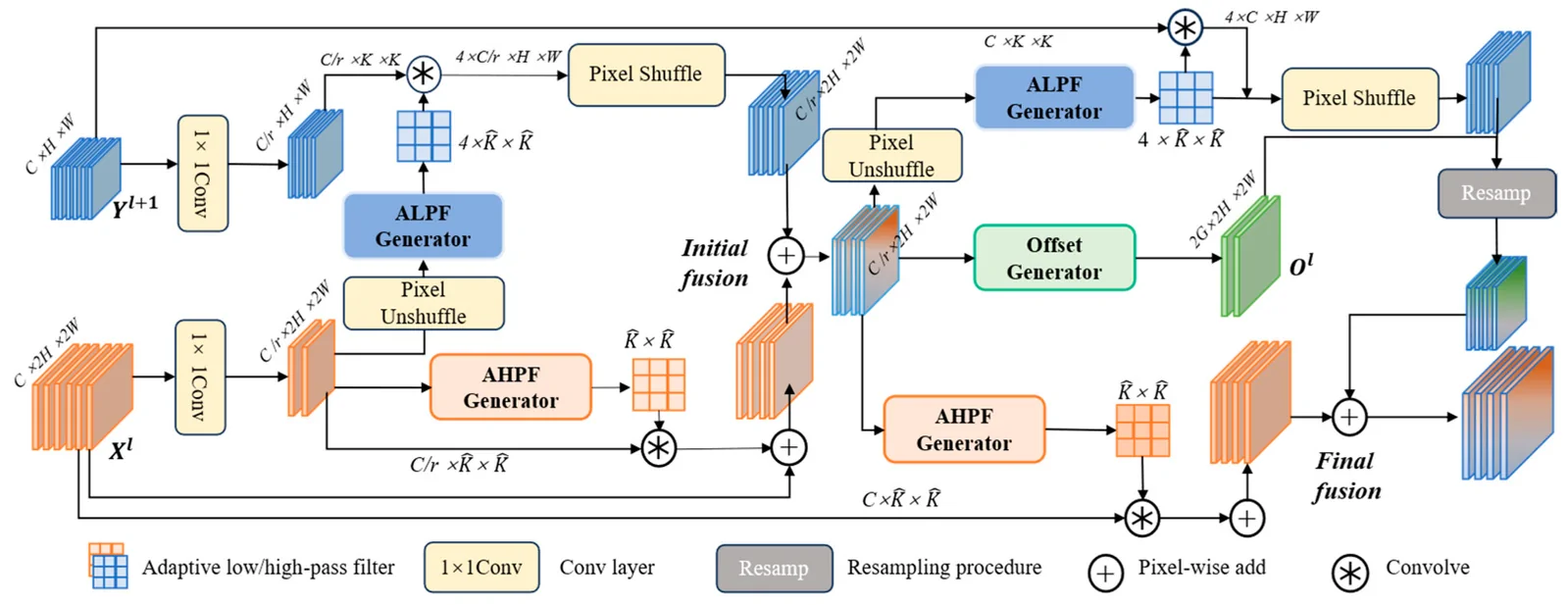
Architecture of the FreqFusion module. Arrows denote the direction of feature flow.

In highway debris target detection, shallow features contain rich edge, texture, and location information, whereas deep features provide stronger semantics but can lose fine details of small targets after repeated downsampling. Multi-scale fusion based solely on concatenation or simple addition may produce unbalanced contributions across scales, insufficient spatial detail, and blurred target boundaries, thereby impairing detection of distant and small debris. To address this issue, we introduce a FreqFusion-BiFPN feature-fusion neck in YO-LO11n [25]. FreqFusion enhances frequency-aware detail and cross-resolution consistency before BiFPN performs bidirectional weighted multi-scale aggregation. The FreqFusion module [26] uses adaptive frequency-modulation to coordinate high-level semantic information and low-level spatial details through frequency-domain decomposition and dynamically generated filters. The specific structure is shown in Figure 2.

The architecture first uses a spatially adaptive low-pass filter (ALPF) to smooth high-level features, thereby eliminating high-frequency interference from background elements such as road clutter and markings. At the same time, a local similarity-oriented feature replacement strategy replaces inconsistent features with resampled class-consistent features to enhance the intra-class consistency of the debris object, thereby alleviating class inconsistency and boundary offset. For low-level features, an adaptive high-pass filter (AHPF) is used to recover high-frequency details lost during downsampling, thereby sharpening the fine edges of bricks, paper boxes, and rocks. In this process, the offset generator dynamically generates a local similarity mask to guide the filtering process of ALPF and AHPF, and aligns the feature spaces through adaptive offset adjustment. Through frequency-domain decomposition with a dynamic weight distribution, the architecture improves debris-boundary preservation while effectively retaining low-level spatial information. For the l-th feature $X^{l}\in R^{C\times 2H\times 2W}$, output from the backbone network and the fused feature $Y^{l+1}\in R^{C\times H\times W}$ from the (l + 1)-th layer, the operations performed by this module on them can be expressed by Equations (1)–(3) [27]:

(1) $${\tilde{Y}}^{l+1}=f_{UP}\left(f_{ALP}\left(Y^{l+1}\right)\right)$$

(2) $${\tilde{X}}^{l}=f_{AHP}\left(X^{l}\right)+X^{l}$$

(3) $$Y_{\mathrm{i},j}^{l}={\tilde{Y}}_{\mathrm{i}+s,j+t}^{l+1}+{\tilde{X}}_{\mathrm{i},j}^{l}$$

Here, $f_{UP}\;$represents the upsampling operation, $f_{ALP}$ represents the low-pass filter predicted by the ALPF generator, $f_{AHP}$ represents the high-pass filter predicted by the AHPF generator, and (*s*, *t*) represents the offset value $Z^{l}$ predicted by the offset generator based on the feature coordinates (*i*, *j*).

After frequency-aware enhancement, BiFPN [28] establishes bidirectional information propagation. The top-down path transfers deep semantic information toward high-resolution features, while the bottom-up path feeds enhanced spatial details back to deeper levels. Unlike unweighted addition, each fusion node assigns learnable weights to its inputs [29]. The weighted fusion process is as follows:

(4) $$O=\sum_{i}\frac{w_{i}}{\epsilon +\Sigma_{j}w_{j}}\cdot I_{i}$$

where $I_{i}$ denotes the multi-scale features input to the fusion node, $w_{i}$ represents the learnable weights assigned to each feature, and ϵ = 0.0001 is a small constant introduced to avoid numerical instability. *O* denotes the fused output feature. This mechanism enables the network to adaptively balance lateral, top-down, and bottom-up information according to their contributions to debris detection, thereby reducing the influence of redundant or irrelevant features.

Overall, the FreqFusion-BiFPN module goes beyond merely stacking FreqFusion and BiFPN. Instead, it first applies frequency-aware enhancement to cross-scale features via FreqFusion, thereby highlighting the edge, texture, and spatial details of debris objects. Then, BiFPN is used to perform bidirectional weighted fusion of the enhanced features, thereby achieving full interaction between shallow detail information and deep semantic information. This module can effectively improve the model’s perception of highway road debris in small-scale, long-distance, and complex backgrounds, and provide richer and more discriminative multi-scale feature representations for subsequent detection heads.

To further disentangle the individual contributions of FreqFusion and BiFPN, a component-level ablation study is presented in Section 4.1.2.

### 2.3. Efficient Multi-Scale Attention Module

**Figure 3:**
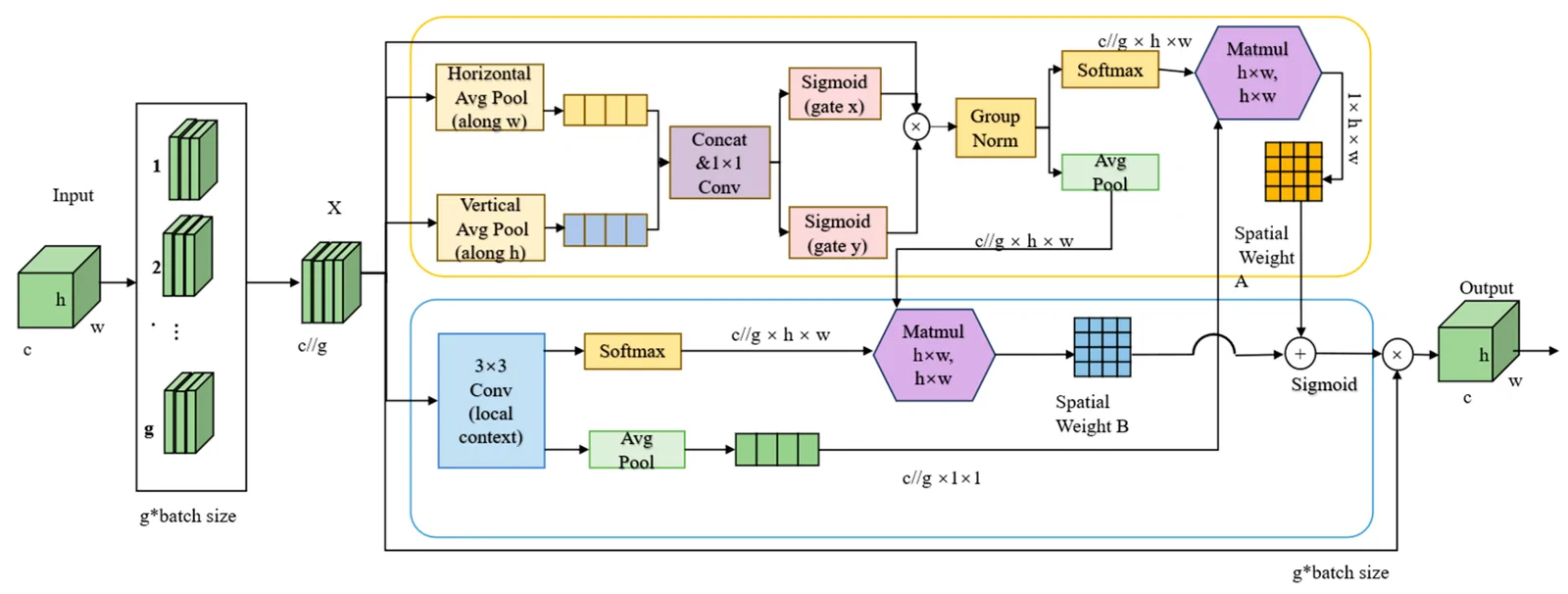
Structure of the EMA module. Arrows denote the direction of feature flow. The asterisk “*” represents multiplication; g*batch size indicates the product of group number g and batch-size dimension.

In FBENet, the EMA module is placed before the P3 detection branch to strengthen the final high-resolution feature representation. This encourages the model to focus more sharply on debris regions, thereby improving small-object detection in complex highway scenes. The architecture of the EMA module is illustrated in Figure 3.

For an input feature map $X\in R^{h\times w\times c}$, the EMA module [30] first divides (X) into (g) subgroups along the channel dimension, denoted as $X=[X_{0},X_{1},\ldots ,X_{g-1}]$, where each subgroup $XX_{i}\in R^{(c/g)\times h\times w}$. This grouping strategy preserves fine-grained channel information while reducing computational complexity, enabling different subgroups to capture complementary semantic representations. Each subgroup is subsequently fed into two parallel branches: in the 1 × 1 branch, spatial positional information is encoded by applying one-dimensional global average pooling along the horizontal and vertical directions. Direction-aware attention weights are then generated through a 1 × 1 convolution followed by a sigmoid activation function; in the 3 × 3 branch, a 3 × 3 convolution is employed to extract local texture and contextual features. The two branches further establish cross-spatial interactions through global average pooling, softmax normalization, and matrix multiplication, thereby integrating directional positional information with local contextual representations to generate a pixel-level spatial attention map. This attention map is multiplied element-wise with the original grouped features, after which all groups are concatenated to reconstruct an output feature map with the same spatial and channel dimensions as the input. For highway road debris detection, EMA enhances the model’s sensitivity to small, weakly textured, and low-contrast objects, such as bricks, paper boxes, and rocks, while suppressing irrelevant responses caused by road textures, shadows, and complex backgrounds. Consequently, the feature discrimination and localization responses for debris objects are enhanced [31]. Since EMA introduces only negligible parameter overhead, it can be seamlessly embedded into the YOLO11n network, providing effective feature enhancement for multi-scale road debris detection in complex road scenes.

## 3. Experiment

### 3.1. Datasets

#### 3.1.1. Synthetic Dataset

Road debris incidents are sporadic, and the large-scale collection of real samples is difficult and can create safety risks. Consequently, publicly available datasets contain relatively few highway debris samples. To alleviate this shortage, this study constructs a road debris detection dataset through image synthesis. Highway images serve as background, and three representative debris types—brick, rock, and paper box—are used as foreground targets. During synthesis, the foreground objects are scaled, repositioned, and blended into the highway scenes. Specifically, foreground objects are resized using a scale factor uniformly sampled from 0.15 to 0.45 relative to the background image width and are placed within the lower half of the image, corresponding to the road region, while ensuring that they do not exceed the image boundaries. Each synthesized image contains one to three debris objects, and the overlap ratio between any two objects is constrained to be below 20% to avoid unrealistic occlusion.

**Figure 4:**
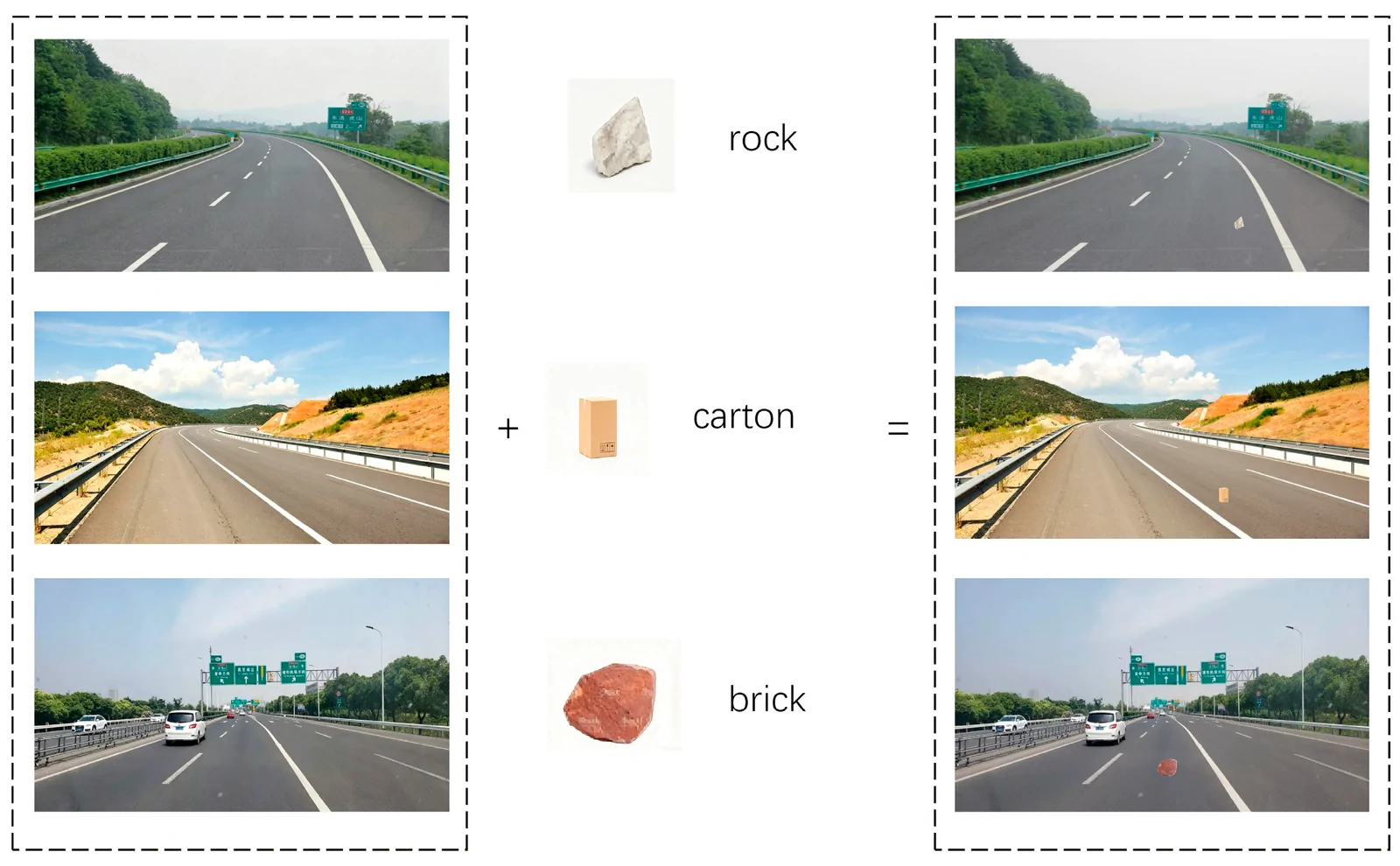
Example of the dataset synthesis process. Dashed boxes represent the original road background images. Each background image is composited with three typical road-debris objects, i.e., rock, paperbox, and brick, to generate synthetic road-scene samples.

Since the road regions were not explicitly annotated with region-of-interest masks, we did not use an automatic random placement strategy that depends on such masks. To improve visual realism, histogram matching was applied to adapt the foreground color distribution to the background, followed by Gaussian boundary smoothing using a kernel size of 3 and random brightness and contrast perturbations within ±15% to simulate different illumination conditions. This approach reduces labeling effort but can occasionally place debris outside realistic road-surface regions [32].To limit this issue, all generated images were manually inspected. Samples with unrealistic object placement, disproportionate scaling, poor blending quality, or annotation inconsistencies were manually discarded, resulting in a curated set of 3966 synthesized images. Figure 4 illustrates the synthesis dataset process.

**Figure 5:**
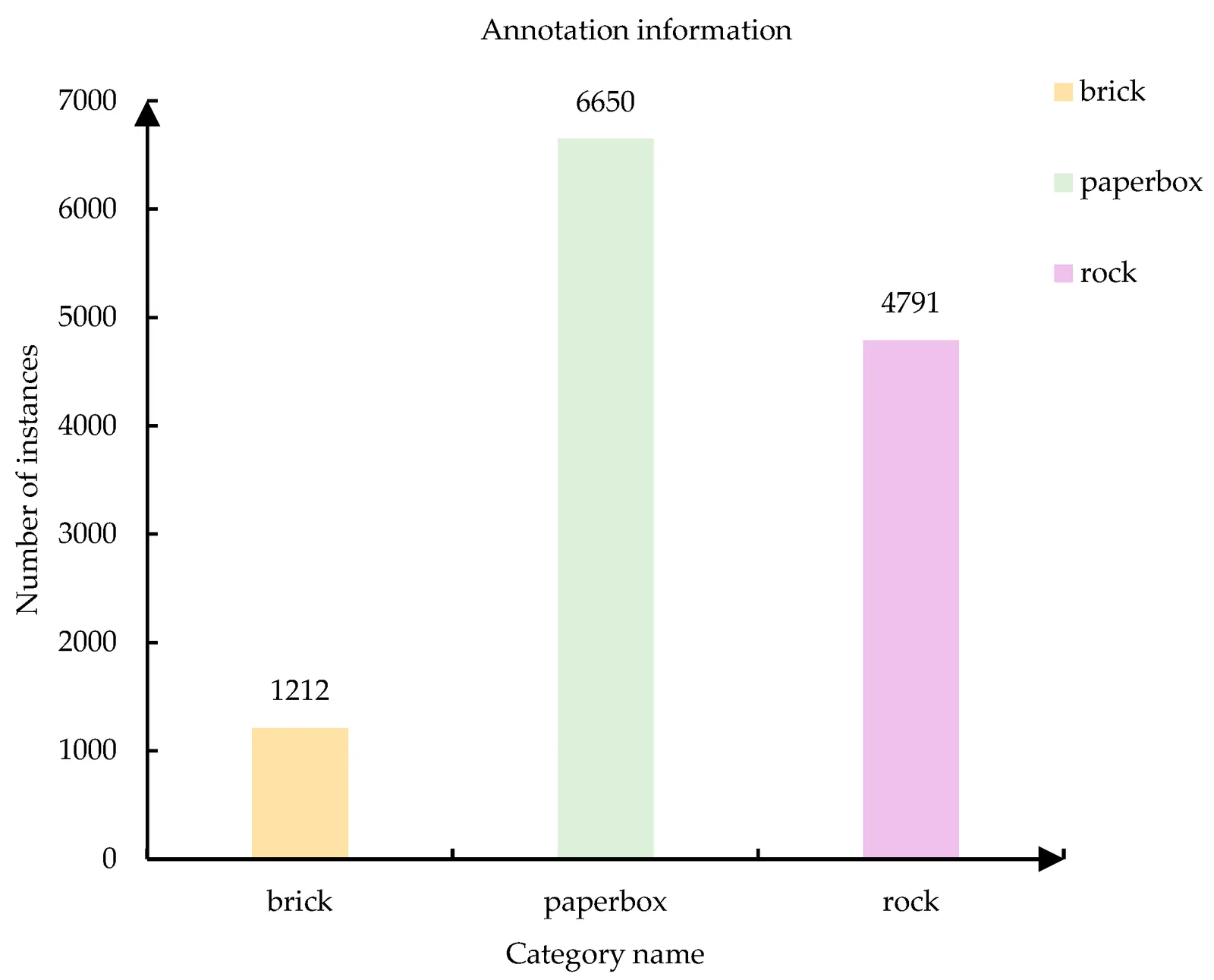
Class distribution of the Synthetic-Debris dataset. The horizontal axis represents category name, and the vertical axis represents number of instances.

All synthesized images were manually annotated using LabelMe, with a tight bounding box assigned to each debris instance. After annotation, an additional manual screening step removed samples containing annotation errors or unrealistic compositing artifacts. Although the synthetic dataset can alleviate the problem of insufficient road litter samples to some extent, it still differs from the real road monitoring scene. To further enrich data diversity, the dataset was expanded to 8433 samples through data augmentation and split into training, validation, and test sets at a ratio of 8:1:1. Through the above processing, the constructed dataset can make up for the defect of insufficient samples of real road throwing objects to a certain extent and provide data support for the training of subsequent target detection models. Brick, paper box, and rock were selected because they represent three common visually distinct debris categories with different shapes, textures, and scales. Other debris types, such as tire fragments and metal parts, may also occur on highways; the current three categories therefore provide a limited benchmark rather than complete coverage of real debris. The synthesis pipeline can be extended to additional categories in future work. The detailed information of the enhanced Synthetic-Debris dataset is shown in Figure 5.

The Synthetic-Debris dataset contains 6650 paper boxes, 4791 rocks, and 1212 brick instances, resulting in a noticeable class imbalance, with brick as the minority category. To evaluate whether this imbalance affects detector performance and the conclusions of this study, we report class-wise detection metrics under the original data distribution and further conduct class-aware sampling experiments. The rebalancing strategy is applied only to the training set, whereas the validation and test sets retain their original natural class distributions. The detailed sampling strategy and experimental results are presented in Section 4.2.

#### 3.1.2. Real-Road Debris Dataset

**Figure 6:**
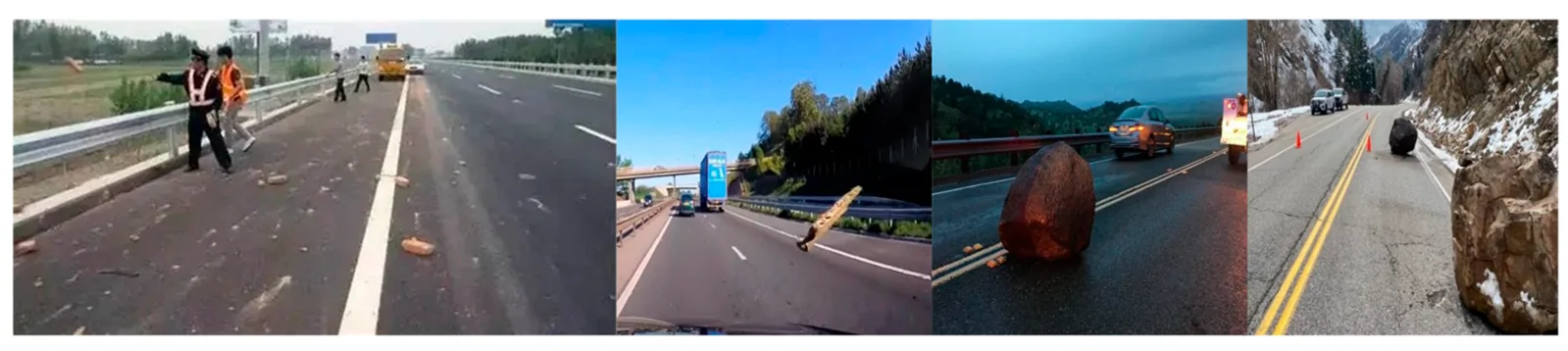
Sample images from the real-road debris dataset.

To evaluate synthetic-to-real transfer under limited real data, we constructed a real-road debris dataset. Images were collected from public image platforms including Google and Baidu using keywords such as road debris, road fragments, and dropped cargo. After manual screening to remove low-quality, duplicate, and irrelevant images, 110 valid images containing 329 annotated debris instances were retained. All annotations were manually performed with the X-AnyLabeling tool. The dataset includes three classes, brick (61 instances), paper box (50 instances), and rock (218 instances), with rocks as the dominant class in this collected set. The fixed training and validation subsets contain 265 and 64 annotated bounding boxes, respectively. Image resolutions range from 324 × 189 to 2048 × 2048 pixels and cover diverse real-world scenes. Given the limited sample size within individual scene types, the dataset was not stratified by scene characteristics. Example images are shown in Figure 6.

### 3.2. Experimental Platform and Parameter Settings

All experiments were carried out on the PyTorch 2.4.1 framework with Python 3.11.15 and CUDA 12.4. The computing platform was equipped with an Intel^®^ Core™ i7-13650HX processor (Intel Corporation, Santa Clara, CA, USA) and an NVIDIA GeForce RTX 4060 (8 GB) graphics card (NVIDIA Corporation, Santa Clara, CA, USA), using local SSD storage on a Windows machine. The specific hyperparameter settings are summarized in Table 2.

The input image resolution size was set to 800 × 800. All models were trained for 200 epochs with the SGD optimizer, an initial learning rate of 0.01, a batch size of 8, and the same augmentation configuration. During inference, the confidence threshold and NMS IoU threshold were fixed at 0.25 and 0.3, respectively.

### 3.3. Evaluation Metrics

To comprehensively evaluate the performance of the debris detection network, this study selects metrics from two dimensions: detection accuracy and model complexity. Detection accuracy is assessed using precision (P), recall (R), F1-score, mAP@0.5, and mAP@0.5:0.95, while model complexity is measured by the number of parameters (Params). For each class, AP@0.5 is calculated as the area under the precision–recall curve at an IoU threshold of 0.5. The reported mAP@0.5 is the arithmetic mean of AP@0.5 over the three debris classes (brick, paper box, and rock). Similarly, mAP@0.5:0.95 is averaged over IoU thresholds from 0.50 to 0.95 with a step size of 0.05.

The F1-Score, defined as the harmonic mean of precision and recall, provides an overall evaluation of the model’s detection performance under a fixed confidence threshold. In thrown-object detection scenarios, missed detections may compromise traffic safety, whereas false detections may increase the false-alarm rate. Therefore, the F1-Score effectively reflects the balance between detection accuracy and completeness. In contrast, mAP provides a more comprehensive evaluation by measuring the trade-off between precision and recall across different IoU thresholds [33]. Higher values of mAP@0.5 and mAP@0.5:0.95 indicate better overall detection performance and stronger localization capability. The formulas for these evaluation metrics are given in Equations (5)–(9):

(5) $$P=\frac{TP}{TP+FP}$$

(6) $$R=\frac{TP}{TP+FN}$$

(7) $$AP=\int_{0}^{1}p\left(r\right)\mathrm{d}r$$

(8) $$mAP=\frac{1}{K}\sum_{k=1}^{K}{AP}_{k}$$

(9) $$F1-Score=\frac{2PR}{P+R}$$

Here, TP, FP, and FN denote the numbers of true positives, false positives, and false negatives, respectively. AP is the area under the precision–recall (P-R) curve, with recall R on the horizontal axis and precision P on the vertical axis.

It should be noted that since the precision–recall (PR) curve generated by object detectors is actually a non-monotonic staircase function composed of discrete confidence thresholds, Equation (7) cannot be directly computed via continuous analytical integration. In practice, following the standard COCO evaluation protocol, we numerically approximate this integral by applying 101-point interpolation over the recall axis, followed by the trapezoidal rule for area estimation. Mathematically, this discrete approximation is expressed as:

(10) $$AP\approx \frac{1}{101}\sum_{r\in (0,0.01,\ldots ,1)}p_{interp}(r)$$

where interp represents interpolated precision.

In addition, class-wise average recall with a maximum of 100 detections per image, denoted as AR@100, is reported in the class-imbalance analysis.

For the runtime benchmarks, all experiments used an input resolution of 800 × 800 and a batch size of 1. The complete test set of 824 images was evaluated in three independent runs after 100 warm-up iterations. The confidence threshold, NMS IoU threshold, and maximum number of detections were fixed at 0.25, 0.7, and 300, respectively. FPS was calculated by dividing the total number of processed images by the total wall-clock end-to-end time.

(11) $$FPS=\frac{N}{T_{wall}}$$

where N is the total number of processed images, and $T_{wall}$ is the total wall-clock time in seconds.

## 4. Experimental Results and Analysis

### 4.1. Ablation Experiment

#### 4.1.1. Module-Level Ablation

To evaluate the contributions of the proposed modules to the YOLO11n baseline, we conducted a series of module-level ablation experiments. FreqFusion-BiFPN and EMA were added individually and jointly, and their effects on detection performance were compared. Table 3 reports the results of the ablation experiment, where “√” indicates that the corresponding module is included.

As shown in Table 3, the proposed improvements enhance the detection performance of YOLO11n on the Synthetic-Debris dataset while preserving a lightweight architecture. The baseline model achieves a precision of 0.851, a recall of 0.719, an F1-score of 0.779, an mAP@0.5 of 0.834, and an mAP@0.5:0.95 of 0.637, with 6.3 GFLOPs and 2.58 M parameters. Its relatively high precision but lower recall indicates that missed detections remain a major limitation in complex highway scenes.

Introducing FreqFusion-BiFPN increases recall to 0.803 and F1-score to 0.813, the highest values among all configurations. Meanwhile, mAP@0.5 and mAP@0.5:0.95 rise to 0.858 and 0.661, respectively. These gains suggest that enhanced multi-scale feature fusion improves the representation of small and low-contrast debris, thereby reducing missed detections. Notably, the parameter count decreases to 2.41 M, although FLOPs increase slightly to 6.6 G.

When EMA is used independently, recall and F1-score increase to 0.772 and 0.803, while mAP@0.5 and mAP@0.5:0.95 reach 0.855 and 0.653. This indicates that EMA strengthens responses to salient debris regions while suppressing background interference. Combining both modules yields the highest mAP@0.5 and mAP@0.5:0.95 values of 0.862 and 0.664. Compared with FreqFusion-BiFPN alone, precision improves from 0.824 to 0.846, whereas recall decreases to 0.779 and F1-score declines slightly to 0.811. The complete model therefore achieves the best overall detection accuracy with only a modest increase in computational cost.

These results indicate that FreqFusion-BiFPN mainly improves sensitivity to debris objects and reduces missed detections, whereas EMA shifts the operating balance toward higher precision and better overall AP. The complete model therefore represents a trade-off between recall and precision rather than a uniform improvement across all metrics.

#### 4.1.2. Component-Level Ablation of the FreqFusion-BiFPN Module

To distinguish the individual roles of FreqFusion and BiFPN and determine whether their combination provides complementary benefits, a component-level ablation experiment was conducted under the same feature levels, channel dimensions, detection heads, dataset split, input resolution, and optimization settings. The results are shown in Table 4.

According to Table 4, applying FreqFusion or BiFPN individually raises the YOLO11n baseline mAP@0.5 from 0.834 to 0.849 and 0.840, respectively. Their joint integration achieves the peak performance (recall: 0.803, F1: 0.813, mAP@0.5: 0.858), demonstrating that the two modules provide complementary benefits through enhanced local frequency perception and cross-scale feature aggregation.

**Figure 7:**
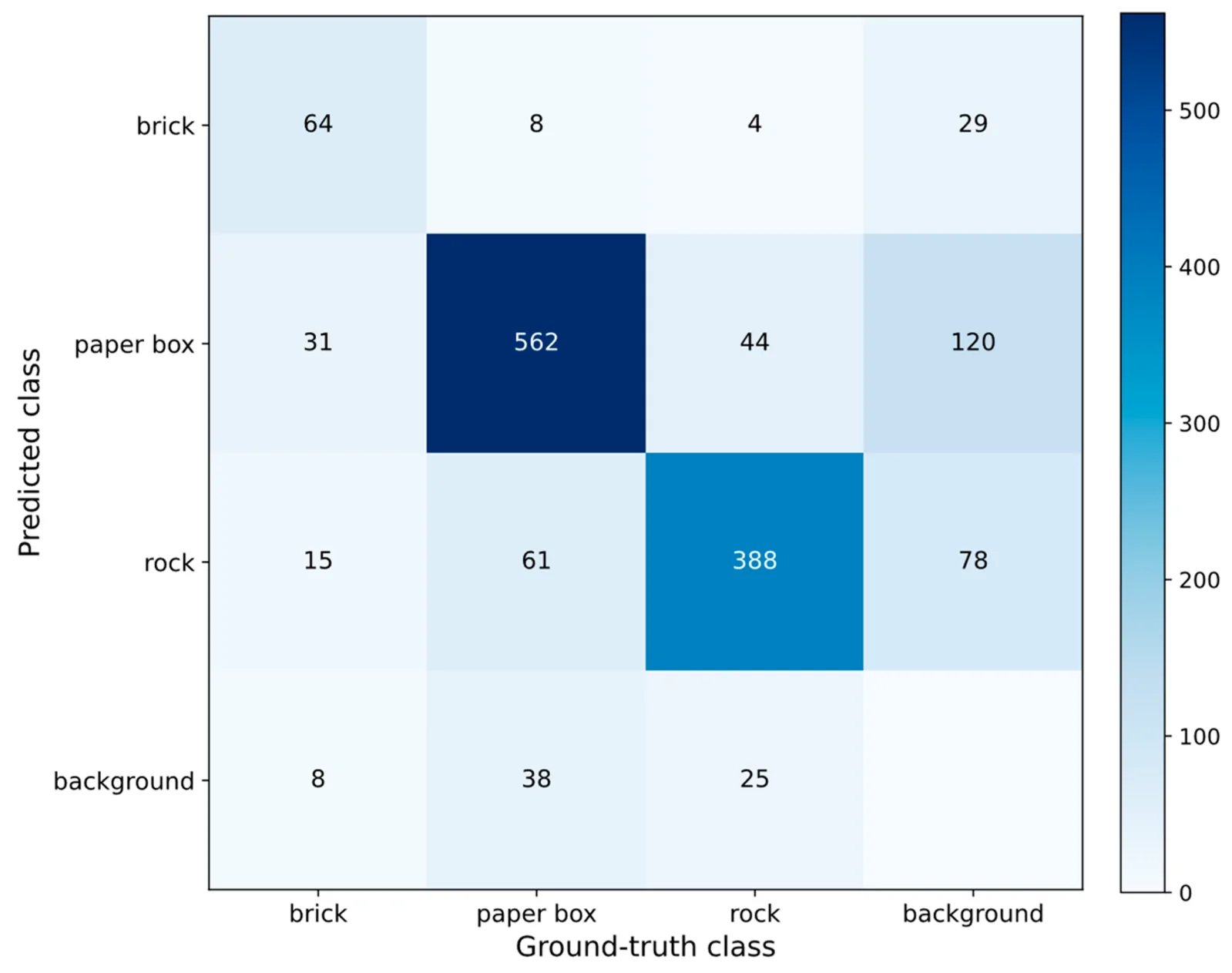
Confusion matrix of FreqFusion-BiFPN. Columns represent ground-truth classes, while rows represent predicted classes. Diagonal cells indicate correct classifications, the background row represents missed detections, and the background column represents false-positive detections.

**Figure 8:**
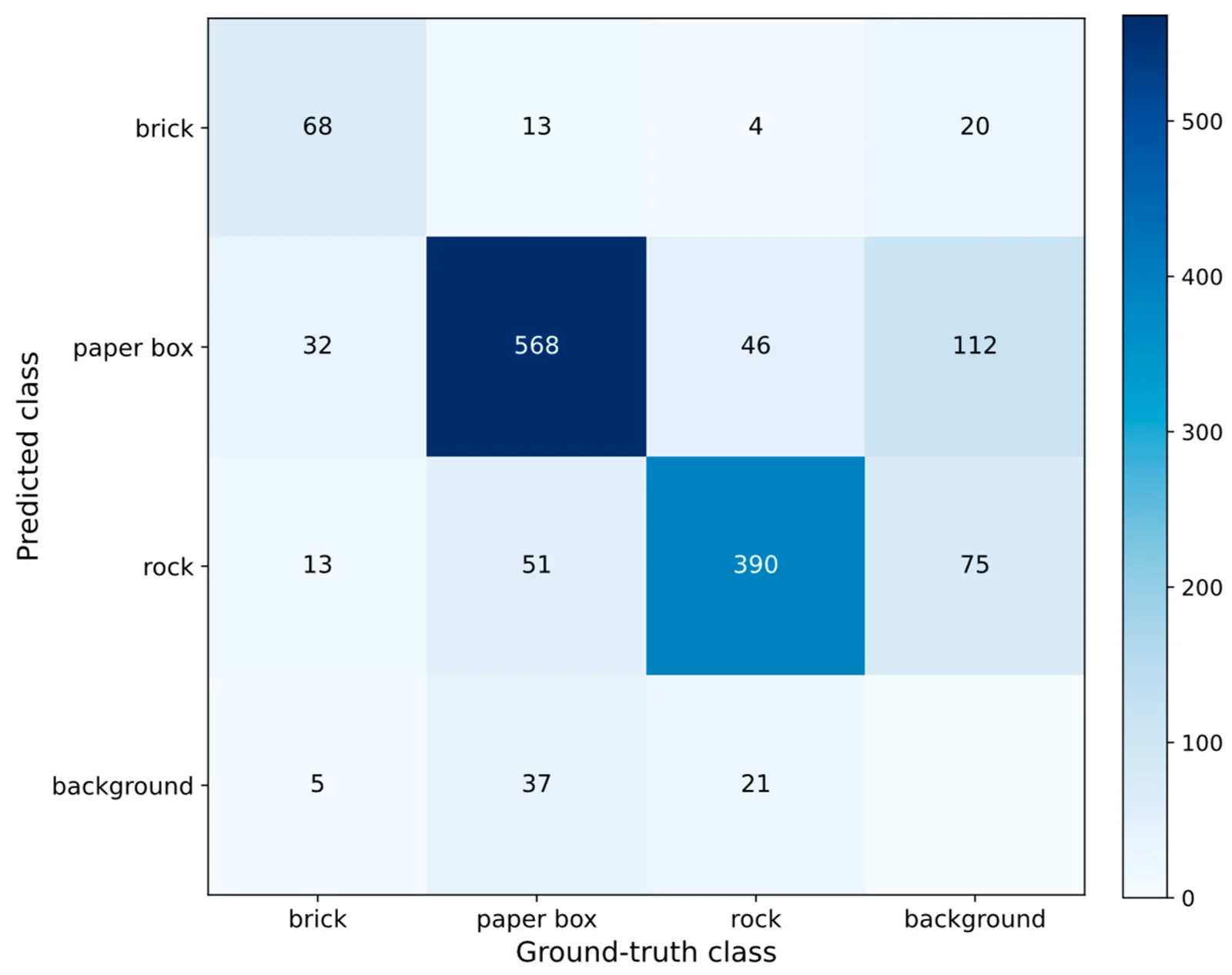
Confusion matrix of EMA. Columns represent ground-truth classes, while rows represent predicted classes. Diagonal cells indicate correct classifications, the background row represents missed detections, and the background column represents false-positive detections.

**Figure 9:**
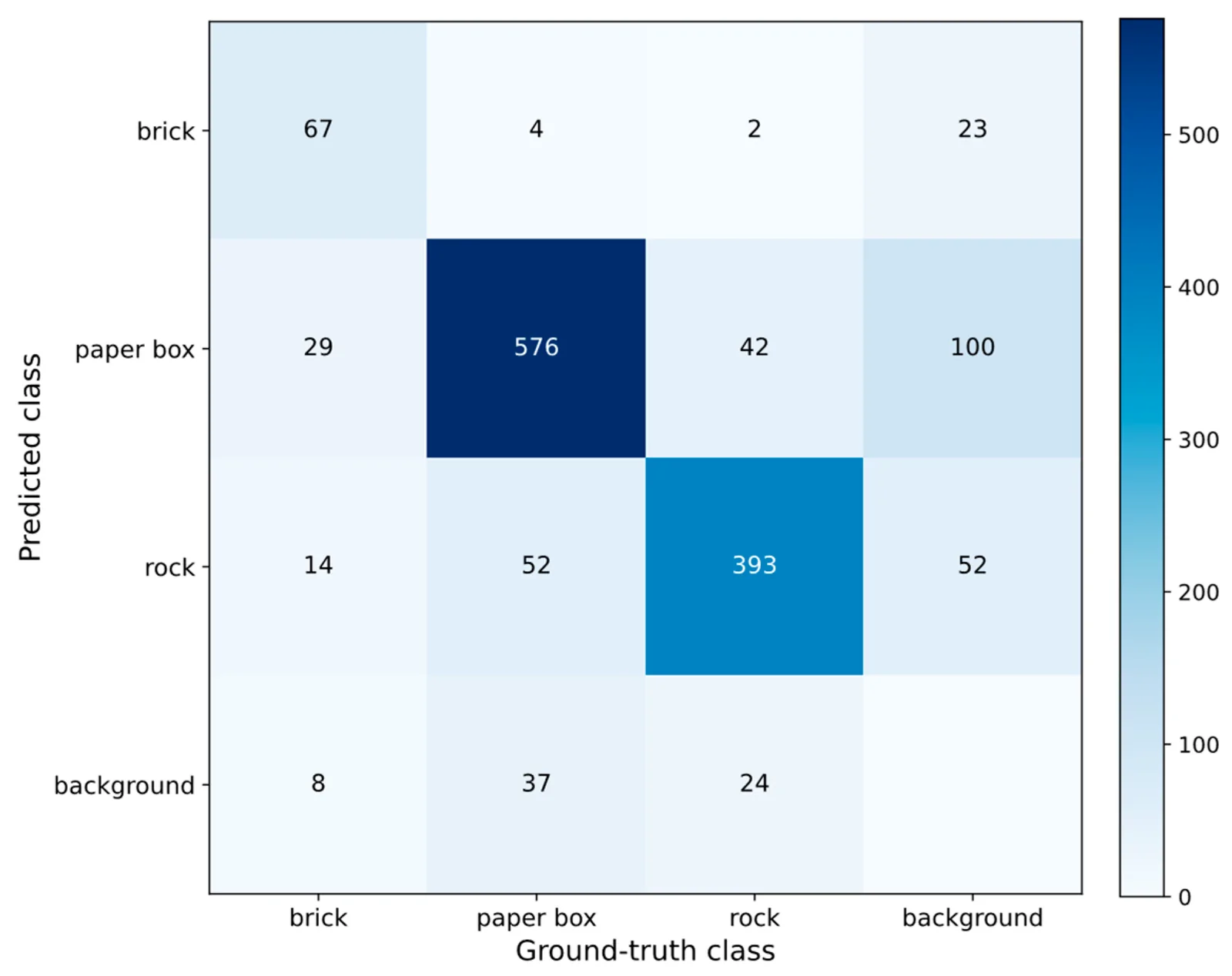
Confusion matrix of FBENet (Ours). Columns represent ground-truth classes, while rows represent predicted classes. Diagonal cells indicate correct classifications, the background row represents missed detections, and the background column represents false-positive detections.

The confusion matrices for FreqFusion-BiFPN, EMA, and FBENet (ours) are presented in Figure 7, Figure 8 and Figure 9, respectively.

### 4.2. Effect of Class Imbalance and Class-Aware Sampling

**Figure 10:**
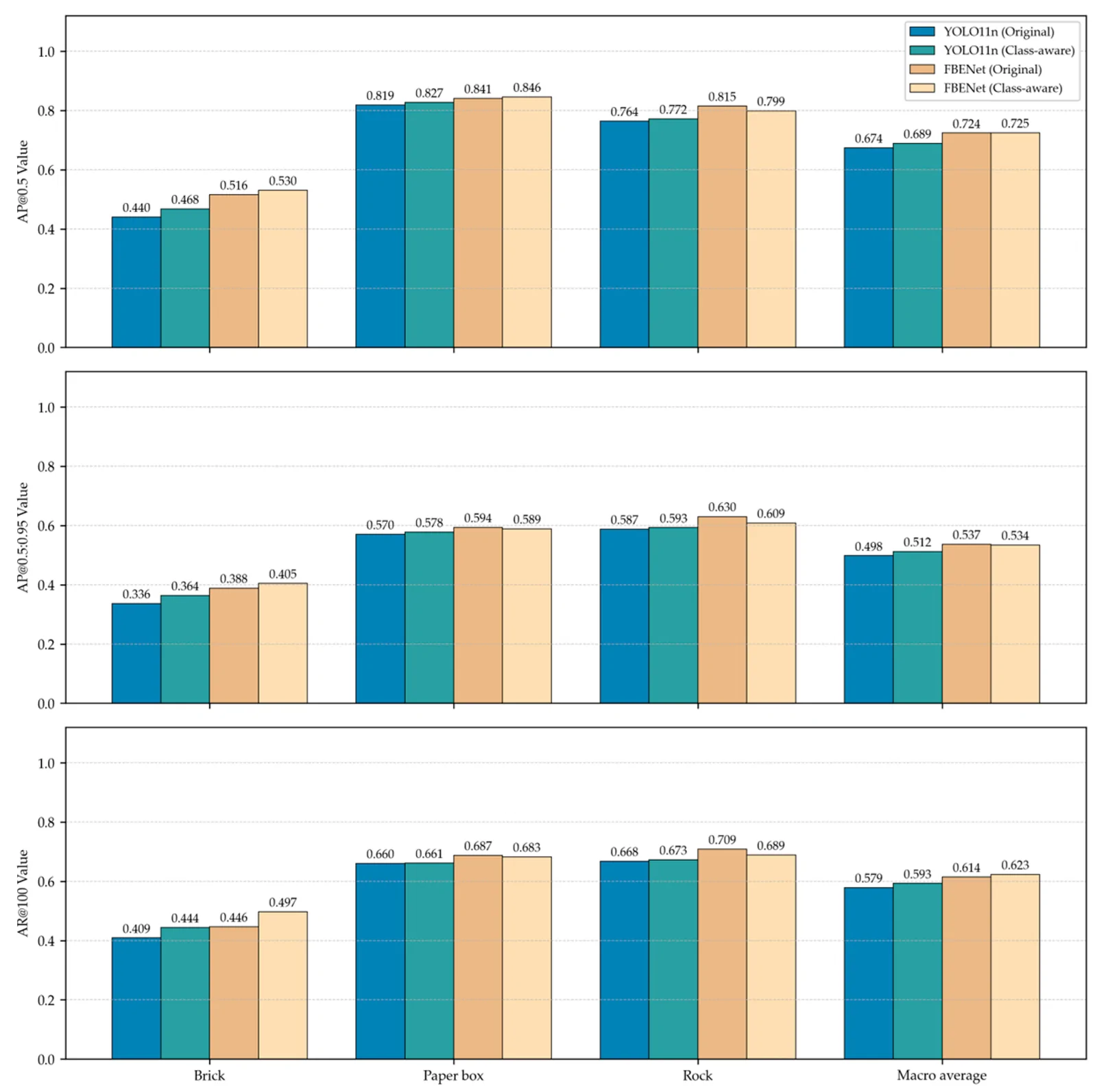
Performance comparison by category and sampling strategy.

We further conducted class-aware sampling experiments for both YOLO11n and FBENet. The strategy is applied only to the training set, while the validation and test sets retain their original natural class distributions. Both models used identical class-aware sampling procedures and training configurations. To keep the number of optimizer updates approximately consistent, the number of training epochs was adjusted from 200 for the original baseline to 159 for the class-aware variants. The results are shown in Figure 10.

As shown in Figure 10, under the original sampling strategy, FBENet consistently outperforms YOLO11n across all three categories. For the minority class brick, FBENet raises AP@0.5 from 0.440 to 0.516, AP@0.5:0.95 from 0.336 to 0.388, and AR@100 from 0.409 to 0.446. Comparable gains are observed for paper box and rock, indicating that FBENet’s improvements are not driven disproportionately by any single majority class.

Under class-aware sampling, which primarily strengthens sensitivity to minority classes, FBENet further improves brick AP@0.5 from 0.516 to 0.530 and AR@100 from 0.446 to 0.497. However, rock exhibits a slight decline (AP@0.5 drops from 0.815 to 0.799), revealing a trade-off between the minority class and other classes rather than uniform gains across categories. Despite this, FBENet maintains a clear advantage over YOLO11n: it achieves a macro AP@0.5 of 0.725, a macro AP@0.5:0.95 of 0.534, and a macroAR@100 of 0.623, compared with 0.689, 0.512, and 0.593 for YOLO11n, respectively. These results indicate that FBENet’s advantage over YOLO11n is not dependent on the original class imbalance.

### 4.3. Runtime and Deployment Performance

To evaluate runtime on the available hardware, all models were tested with a batch size of 1 on an RTX 4060 Laptop GPU. The PyTorch FP32 experiments used the same input resolution, confidence threshold, NMS IoU threshold, maximum number of detections, image sequence, and warm-up protocol. Preprocessing, network inference, post-processing, mean wall-clock end-to-end latency, P99 latency, and FPS were recorded. Specifically, at an input resolution of 800 × 800 with a batch size of 1. Detailed results are presented in Table 5.

As shown in Table 5, the additional feature-fusion and attention operations introduce measurable runtime overhead under PyTorch FP32. Compared with YOLO11n, FBENet increases the network inference latency from 8.07 to 12.79 ms, corresponding to an increase of 58.5%. The mean end-to-end latency increases from 12.63 to 18.53 ms (+46.7%), while throughput decreases from 79.15 to 53.97 FPS (−31.8%). The P99 latency also increases from 20.17 to 36.75 ms. Therefore, FBENet does not improve inference speed relative to the YOLO11n baseline under the PyTorch FP32 setting.

The intermediate variants show that both proposed components contribute to the runtime increase. YOLO11n with FreqFusion-BiFPN achieves 67.55 FPS, whereas YOLO11n with EMA achieves 68.01 FPS. Their combination in FBENet further reduces the throughput to 53.97 FPS. These results demonstrate that the improved detection performance is accompanied by additional computational and execution overhead.

For optimized deployment evaluation, YOLO11n and FBENet were further converted into TensorRT FP16 engines. As shown in Table 6, FBENet achieves a mean end-to-end latency of 7.90 ms, a P99 latency of 10.78 ms, and a throughput of 126.59 FPS.

Under TensorRT FP16, FBENet is slower than YOLO11n (126.59 versus 137.64 FPS) but improves mAP@0.5 from 0.834 to 0.862. Relative to its PyTorch FP32 runtime, TensorRT reduces FBENet’s mean end-to-end latency from 18.53 to 7.90 ms and increases throughput from 53.97 to 126.59 FPS. The measured throughput indicates that FBENet can process more than 100 images per second under TensorRT FP16 on the evaluated GPU. However, whether this satisfies real-time requirements depends on the target frame rate, the complete perception pipeline, and the deployment hardware.

### 4.4. Evaluation and Ablation of Attention Mechanisms

#### 4.4.1. Comparison of Different Attention Mechanisms

To further analyze the impact of different attention mechanisms on the benchmark model, we replaced the EMA module with other popular attention mechanisms at the P3 position in the Neck of YOLO11n, and compared their performance. The results are shown in Table 7.

Table 7 compares different attention mechanisms inserted at the same P3 position of YOLO11n. Under the same training and evaluation settings, the baseline YOLO11n achieves an mAP@0.5 of 0.834. After introducing EMA, the mAP@0.5 increases to 0.855, corresponding to an improvement of 2.1 percentage points. Meanwhile, the FLOPs increase only slightly from 6.3 to 6.4 G, while the parameter count remains approximately unchanged at 2.58 M.

ECA-YOLO11n achieves an mAP@0.5 of 0.841, representing a modest improvement over the baseline. In contrast, SimAM-YOLO11n achieves an mAP@0.5 of 0.826 and does not improve detection performance under the current experimental setting. Therefore, EMA was selected for the final FBENet architecture because it achieves the highest detection accuracy among the evaluated attention mechanisms while introducing only marginal additional computational cost.

**Figure 11:**
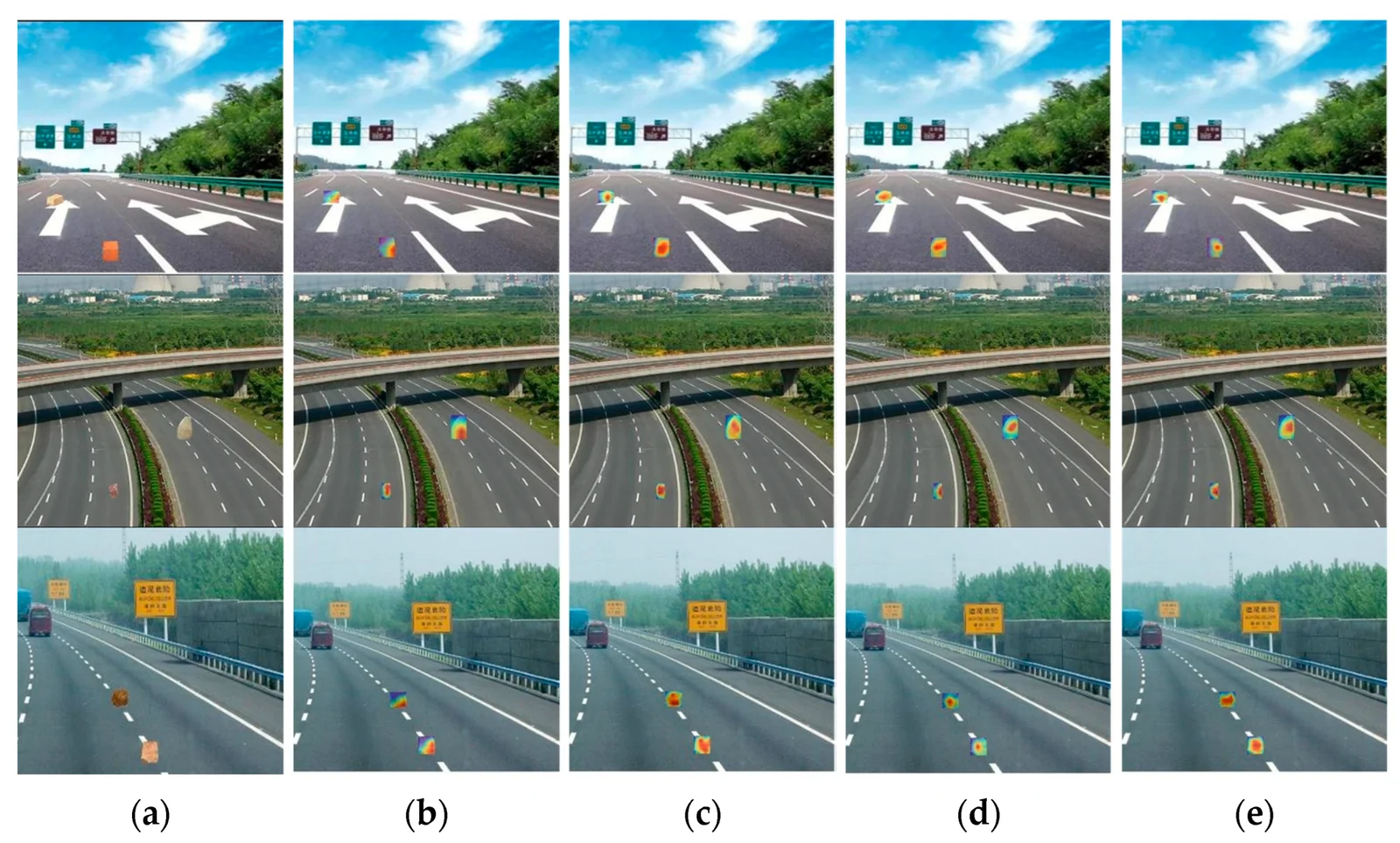
Feature-map comparison for different attention mechanisms. (**a**) Original image. (**b**) YOLO11n. (**c**) EMA-YOLO11n (Efficient Multi-scale Attention). (**d**) ECA-YOLO11n (Efficient Channel Attention). (**e**) SimAM-YOLO11n (Simple, Parameter-free Attention Module). Red indicates high activation where the model focuses most, yellow represents moderate activation, and blue denotes low-activation regions with little contribution to detection.

Figure 11 visualizes the feature maps produced with different attention mechanisms. In the selected example, EMA produces stronger responses in regions associated with the target objects.

#### 4.4.2. Ablation of EMA Placement in the Feature Pyramid

To investigate the impact of EMA placement within the feature pyramid, we evaluated five placement strategies: P3, P4, P5, P3 + P4, and P3 + P4 + P5. The results are presented in Table 8.

As shown in Table 8, placing EMA only at P3 achieves the best overall detection performance, with an mAP@0.5 of 0.855 and an mAP@0.5:0.95 of 0.653. In comparison, placing EMA only at P4 or P5 results in mAP@0.5:0.95 values of 0.630 and 0.643, respectively. Extending EMA from P3 to P3 + P4 decreases mAP@0.5:0.95 from 0.653 to 0.635, corresponding to a reduction of 1.8 percentage points. Applying EMA to all three feature levels, P3 + P4 + P5, also yields an mAP@0.5:0.95 of 0.635 and does not provide any additional performance gain.

These results demonstrate that the performance improvement mainly originates from enhancing the P3 feature level rather than from applying EMA throughout the entire feature pyramid. Therefore, EMA is applied only to P3 in the final model. This placement achieves the highest detection accuracy while avoiding unnecessary attention operations at multiple feature levels, providing the best trade-off between detection performance and computational efficiency.

### 4.5. Comparative Experiments of Different Models

#### 4.5.1. Performance Analysis of Different Models

To comprehensively evaluate the competitiveness of FBENet, we compared it with representative detectors from different categories, including mainstream YOLO-series lightweight detectors, a Transformer-based detector, road-debris- or road-object-related methods, and deployment-oriented lightweight variants. These baselines include RT-DETR, YOLOv8n, YOLOv10n, YOLO11n, YOLO12n, YOLOX, ADWNet, CIEFRNet, RTMDet-tiny, YOLO11n-DySample-P2, and YOLO12-FreqFusion-EMA. The comparative results are summarized in Table 9.

Table 9 indicates that FBENet provides the strongest overall performance on the Synthetic-Debris dataset, although it does not lead every individual metric. It achieves a precision of 0.846, recall of 0.779, mAP@0.5 of 0.862, mAP@0.5:0.95 of 0.664, and an F1-score of 0.811. FBENet ranks first in mAP@0.5, mAP@0.5:0.95, and F1-score, while its precision ranks second, only 0.004 behind YOLO11n. Although its recall falls below those of YOLO12n and RTMDet-tiny by 1.5 and 0.5 percentage points, respectively, this is offset by notably higher precision and overall accuracy. These results indicate that FBENet’s main strength is balanced detection rather than optimization of a single metric. It also retains a compact size of 2.43 M parameters and a computational cost of 6.8 GFLOPs.

**Figure 12:**
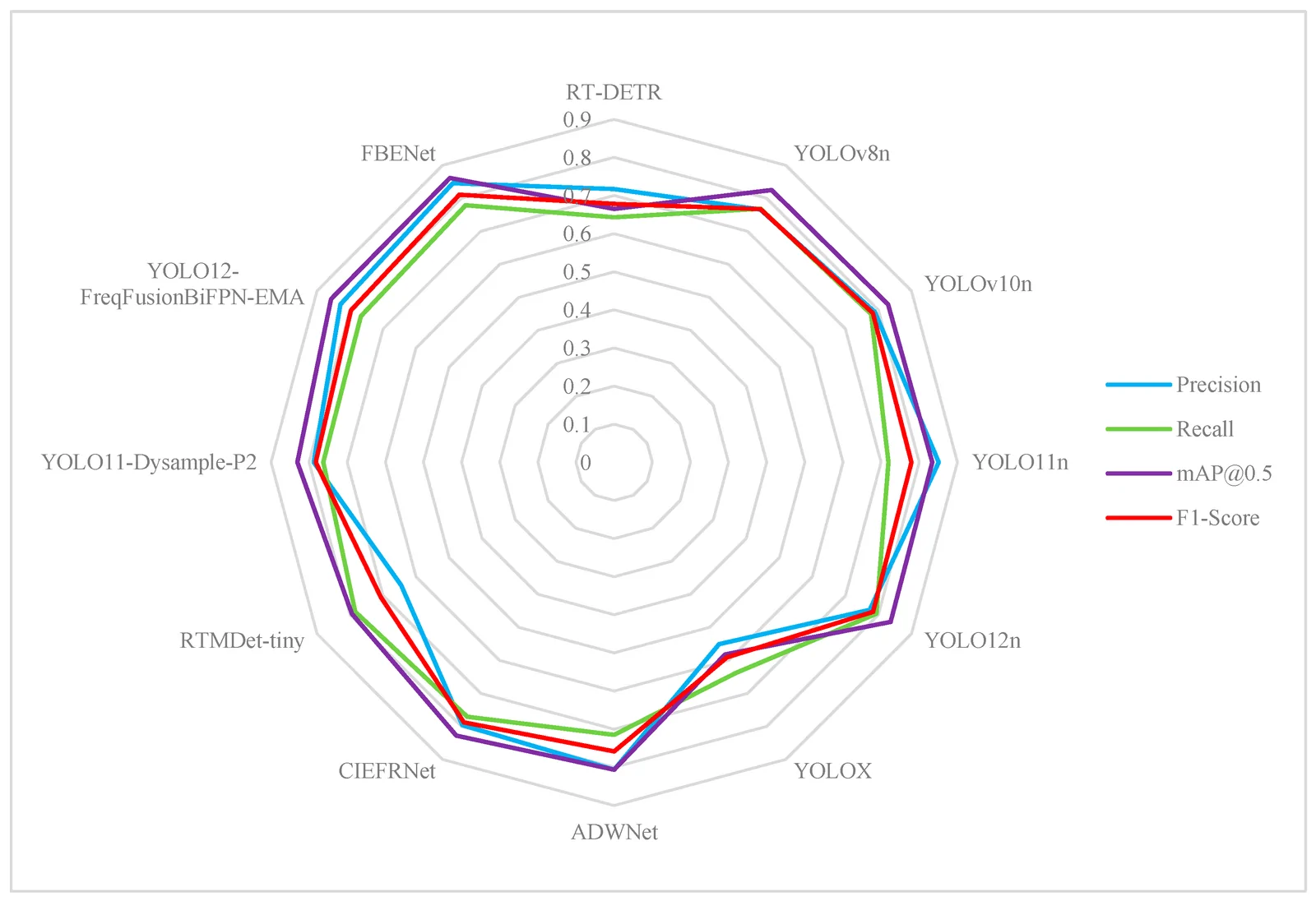
Comparison of precision, recall, mAP@0.5, and F1-score across models.

As shown in Figure 12, FBENet (Ours) forms one of the largest and most balanced radar profiles across precision, recall, mAP@0.5, and F1-score. Compared with YOLOv8n, YOLOv10n, YOLO11n, and YOLO12n, FBENet improves mAP@0.5 by 3.7, 3.3, 2.8, and 2.5 percentage points, respectively, and F1-score by 4.4, 2.8, 3.2, and 2.7 percentage points. Thus, the gains are distributed across multiple criteria rather than being concentrated in a single metric.

The precision and recall comparison further illustrates this balance. YOLO11n attains the highest precision of 0.851, slightly above FBENet’s 0.846, but its recall is lower at 0.719. YOLO12n shows the opposite pattern: with a recall of 0.794 and precision of 0.774. RTMDet-tiny has a recall of 0.784 but a precision of only 0.645. In contrast, FBENet maintains a recall of 0.779 together with strong precision, resulting in the highest F1-score of 0.811 among the compared models. This balance is relevant to highway debris detection, where both missed objects and false alarms affect practical reliability.

Compared with RT-DETR, YOLOX, ADWNet, and CIEFRNet, FBENet also demonstrates more stable performance across the four metrics. The radar curves of RT-DETR and YOLOX remain closer to the chart center, especially for YOLOX, reflecting weaker localization and recognition capability. Although ADWNet and CIEFRNet achieve competitive results on individual indicators, their overall curve expansion remains below that of FBENet. Relative to CIEFRNet, FBENet improves mAP@0.5 and F1-score by 3.4 and 2.3 percentage points, respectively, while using substantially fewer parameters.

FBENet also retains clear advantages over the two improved baselines, YOLO11-DySample-P2 and YOLO12-FreqFusion-EMA. Its mAP@0.5 is higher by 3.1 and 0.5 percentage points, respectively, with consistently better or more balanced precision, recall, and F1-score. In particular, the outward shift of all four curves relative to YOLO12-FreqFusion-EMA suggests that the proposed architecture strengthens feature representation for small debris, scale variation, and cluttered backgrounds.

Overall, Figure 12 shows that FBENet’s gains are distributed across multiple metrics rather than concentrated in a single indicator. By combining high precision, competitive recall, and the best mAP@0.5 and F1-score among the compared models, FBENet provides a favorable balance between false positives and missed detections on Synthetic-Debris.

#### 4.5.2. P-R Curve Analysis

**Figure 13:**
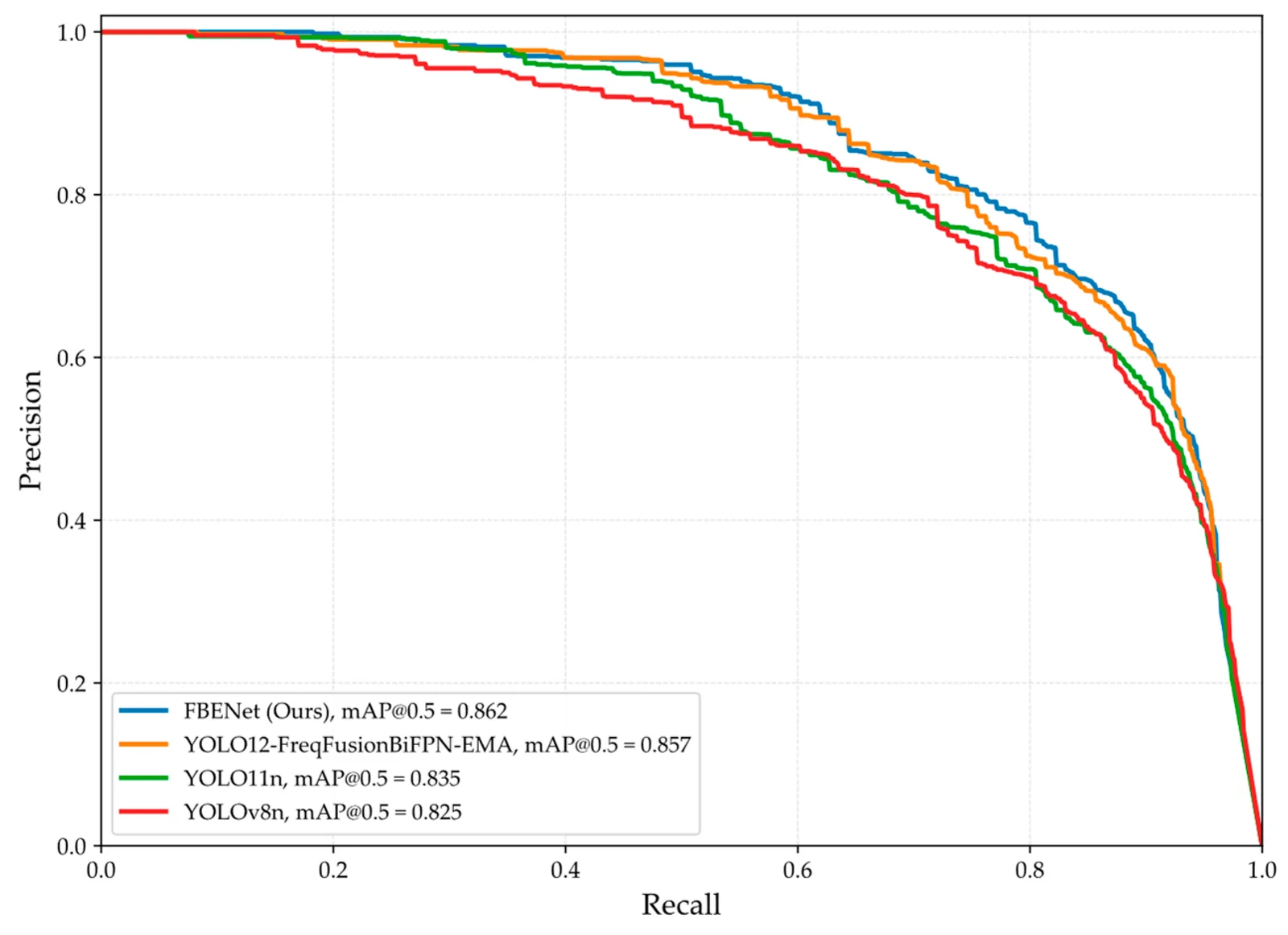
Comparison of P-R curves of different models.

This study further examines the precision–recall curves of several models, including YOLOv8n, YOLO12-FreqFusion-BiFPN-EMA, YOLO11n, and FBENet (Ours), as shown in Figure 13.

Figure 13 further confirms the comprehensive advantages of FBENet (Ours) for highway debris detection, as shown by the precision–recall curves of different models. Compared with YOLOv8n, YOLO11n, and YOLO12-FreqFusion-BiFPN-EMA, FBENet’s P-R curve lies closer to the upper-right corner of the coordinate system, indicating that it maintains high precision across various recall levels. Notably, in the medium-to-high recall range, FBENet’s curve declines more gently, suggesting that the model improves recall without substantially increasing the risk of false detections. This reflects a more stable precision–recall trade-off.

According to the AP results, FBENet (Ours) achieved an AP of 0.921, which was higher than 0.914, 0.905, and 0.901 for YOLO12-FreqFusion-BiFPN-EMA, YOLO11n, and YOLOv8. The results indicate that FBENet’s performance improvement is reflected not only in the single-point index at a fixed threshold but also in improved detection stability across the full confidence range. Based on the indicators in the table and the radar chart results, FBENet (Ours) exhibits a more balanced overall performance across precision, recall, mAP@0.5, and F1-Score, and is better suited to debris target detection tasks in complex highway scenarios.

### 4.6. Visualization Results and Analysis

**Figure 14:**
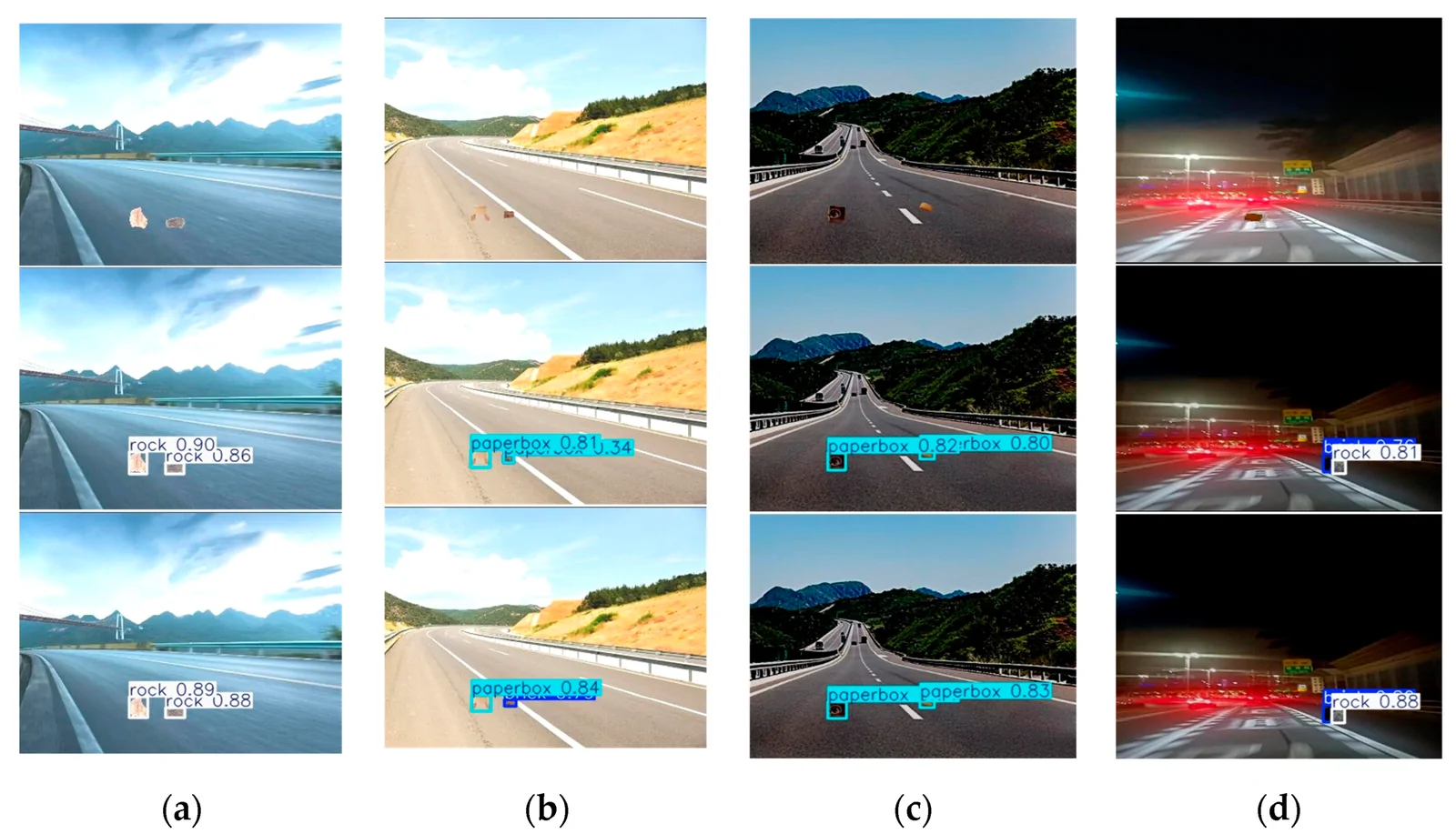
Visualization results for YOLO11n and FBENet models: (**a**) Dawn. (**b**) Daytime. (**c**) Dusk. (**d**) Nighttime. White bounding boxes represent the rock category, light-blue bounding boxes represent the paperbox category, and dark-blue bounding boxes represent the brick category.

To intuitively assess the performance of the improved model against the baseline in complex scenes, test images covering four typical lighting conditions—dawn, daytime, dusk, and nighttime—were selected for a qualitative comparison with YOLO11n. The results are shown in Figure 14. In the figure, the first row displays the original images, the second row presents the detection results of YOLO11n, and the third row shows those of FBENet (Ours). The predicted object categories, bounding boxes, and confidence scores are annotated directly in the figure.

From the perspective of category analysis and confidence performance, FBENet (ours) demonstrates stronger target recognition across different scenarios. In the daytime scene in Figure 14b, YOLO11n assigns only 0.34 confidence to the target on the right and misclassifies it, mistaking a brick for a paper box. By contrast, FBENet (Ours) assigns a confidence of 0.84 to the same target and correctly identifies its category, suggesting that the improved model learns more appropriate feature representations for small objects. In the nighttime scene in Figure 14d, the image is affected by low illumination, motion blur, and vehicle-light interference, significantly increasing detection difficulty. Although both models can detect the target, FBENet’s prediction confidence for the near rock target increases from 0.81 in YOLO11n to 0.88, indicating that, in the selected sample, FBENet (ours) still shows strong target-response ability under weak-light and blur conditions. Furthermore, in the well-lit scene of Figure 14a, FBENet (ours) predicts the two rock targets with confidence scores of 0.89 and 0.88, respectively, indicating a more balanced numerical distribution. This reflects good consistency and stability in responding to similar targets.

In terms of detection redundancy suppression, FBENet (ours) effectively reduces repeated detections. In the dusk scene of Figure 14c, YOLO11n produces an overlapping detection box for the left paper box target, indicating scattered target responses under complex illumination and long-range, small-object conditions, resulting in redundant detections. By contrast, FBENet outputs only one bounding box for the target, yielding more concise and stable detection results, indicating that the improved feature extraction and fusion mechanism enhance the concentration of the target-area response.

Comprehensive visualization results show that FBENet (ours) has strong target detection performance across different lighting conditions, including daytime, dusk and nighttime, particularly in complex scenes characterized by small targets, low illumination and motion blur. It demonstrates higher detection confidence and a lower tendency towards redundant detections. Compared with YOLO11n, FBENet (ours) shows advantages in category discrimination, confidence calibration, and suppression of duplicate boxes, indicating that the proposed improvement strategy can effectively enhance the feature representation of road foreign-object targets and improve the model’s detection robustness in complex traffic environments. This conclusion is consistent with the previous quantitative performance analysis results.

### 4.7. Synthetic-to-Real Transfer Evaluation

#### 4.7.1. Fixed-Split Evaluation

To preliminarily evaluate synthetic-to-real transfer, the 110 real-road debris images were first divided into a fixed split of 90 images for fine-tuning and 20 images for testing. YOLO11n and FBENet were initialized from their corresponding Synthetic-Debris pre-trained weights and fine-tuned using the same real-data protocol to enable a controlled comparison.

As shown in Table 10, the fixed 90/20 split shows higher mAP@0.5 and mAP@0.5:0.95 for FBENet (Ours) than for YOLO11n. Class-wise, AP values also increases for brick, paper box, and rock in this split. These results provide preliminary evidence that the proposed feature pathway can transfer to the limited real-road dataset after fine-tuning, but the small test set does not support broad claims of real-world generalization.

#### 4.7.2. Five-Fold Cross-Validation

To further evaluate the synthetic-to-real transfer ability of the models under limited real-scene samples, we conducted five-fold cross-validation on the 110 real highway debris images. In each fold, approximately four-fifths of the real images were used for fine-tuning, and the remaining one-fifth was used for validation. Both YOLO11n and FBENet were initialized from their corresponding Synthetic-Debris pretrained weights and trained using the same hyperparameter settings. For each fold, AP@0.5 and AP@0.5:0.95 were calculated for brick, paper box, and rock, and the mean, standard deviation, and variance across the five folds were reported. Since mAP is defined as the mean AP over all categories, class-wise results are reported as AP, while the overall row represents the mean performance over all classes. The mean, standard deviation, and variance across the five folds are now provided in Table 11.

As shown in Table 11, FBENet achieved an overall AP@0.5 of 0.420 ± 0.077 and AP@0.5:0.95 of 0.226 ± 0.049, slightly higher than the 0.412 ± 0.089 and 0.213 ± 0.055 obtained by YOLO11n. Moreover, the variance of overall AP@0.5 decreased from 0.0080 to 0.0059, and the variance of overall AP@0.5:0.95 decreased from 0.0030 to 0.0024, suggesting that FBENet provides more stable overall transfer performance across folds. At the class level, the improvement was most evident for brick, where AP@0.5 increased from 0.414 to 0.465 and AP@0.5:0.95 increased from 0.258 to 0.291. For paper box, FBENet obtained slightly higher AP values than YOLO11n, although its recall was slightly lower. For rock, AP@0.5 is lower, AP@0.5:0.95 is comparable, and recall is slightly higher than with YOLO11n.

These five-fold cross-validation results indicate overall synthetic-to-real gains for FBENet, but the effects are class-dependent. The observed variation is likely influenced by the limited number of real samples, class imbalance, and differences in object appearance.

Overall, the five-fold results provide a more explicit and statistically supported view of synthetic-to-real transfer than the single fixed split. It suggests a small improvement in mean overall AP and lower overall AP variance while also showing that FBENet is not uniformly superior for every class.

#### 4.7.3. Class-Wise Transfer Analysis

To further evaluate the error patterns and limitations of FBENet (Ours) in the fixed real-scene validation split, we conducted a class-wise transfer analysis on the fixed real-scene validation set. The results are presented in Table 12.

As shown in Table 12, under the fixed evaluation threshold, FBENet reduces the overall miss rate from 31.3% to 23.4% and increases overall recall from 68.8% to 76.6%. The normalized false-positive ratio was also reduced from 15.6% to 7.8%. At the class level, the miss rate of brick decreased from 37.5% to 25.0%, paper box decreased from 40.0% to 20.0%, and rock decreased from 29.4% to 23.5%. Meanwhile, the normalized false-positive ratio decreased from 12.5% to 0% for brick, from 20.0% to 0% for paper box, and from 15.7% to 9.8% for rock.

On this fixed split, FBENet therefore improves both missed-detection and false-positive behavior. Because these statistics are based on only 20 test images and 64 ground-truth instances, they should be interpreted as split-specific evidence rather than as proof of broad real-scene robustness.

## 5. Discussion

This study addresses limited real-world training data, weak small-target features, and strong background interference in highway debris detection by constructing Synthetic-Debris and developing FBENet on the YOLO11n baseline. The results indicate that the stage-coupled combination of frequency-aware cross-scale enhancement, bidirectional aggregation, and selective P3 attention improves detection on the synthetic benchmark and provides preliminary synthetic-to-real benefits after limited real-data fine-tuning. The technical contribution lies in the task-oriented organization of established modules rather than in proposing new primitive operators.

Ablation experiments show that the FreqFusion-BiFPN and EMA contribute differently. FreqFusion-BiFPN increases mAP@0.5 from 0.834 to 0.858 and recall from 0.719 to 0.803. This suggests that frequency-aware detail enhancement and bidirectional fusion improve sensitivity to small and low-contrast debris. EMA alone increases mAP@0.5 to 0.855. When EMA is combined with FreqFusion-BiFPN, mAP@0.5 increases further to 0.862 and precision increases from 0.824 to 0.846. Thus, the complete model does not improve every metric simultaneously; it shifts the precision–recall balance toward higher precision and the best AP-based accuracy. Given the small F1-score difference, the superiority of either configuration in terms of F1 should not be overinterpreted without repeated experiments.

Compared with mainstream object detectors, FBENet (ours) achieves superior overall performance on the Synthetic-Debris dataset. Its mAP@0.5 and F1-score reach 0.862 and 0.811, respectively, surpassing YOLOv8n, YOLOv10n, YOLO11n, YOLO12n, and other methods. Relative to YOLO11n, FBENet (ours) improves both recall and F1-score while maintaining a low parameter count and modest computational cost, indicating that the proposed architecture does not simply chase higher accuracy; rather, it strikes a more balanced trade-off among missed-detection control, false-positive suppression, and model complexity. The P-R curve and radar chart further demonstrate that FBENet performs more consistently across multiple evaluation metrics. This property is practically meaningful for safety-critical tasks such as highway debris detection, where missing a target carries a particularly high cost.

The synthetic-to-real experiments provide preliminary evidence that the proposed feature pathway transfers beyond the synthetic domain. In the fixed split, FBENet increases mAP@0.5 from 0.598 to 0.648 and mAP@0.5:0.95 from 0.259 to 0.287. Five-fold cross-validation yields smaller mean gains, from 0.412 to 0.420 in AP@0.5 and from 0.213 to 0.226 in AP@0.5:0.95, together with lower overall AP variance. The class-wise effects are not uniform: brick improves most clearly; paper box shows slightly higher AP but slightly lower recall; and rock shows lower AP@0.5, comparable AP@0.5:0.95, and slightly higher recall. The transfer results should therefore be interpreted as an overall tendency under limited real data rather than consistent superiority for every category and split.

The real-scene evaluation should be interpreted with caution because it is based on a relatively small dataset comprising 110 images and 329 annotated instances. On the fixed 20-image test split, the recall improvement is accompanied by a reduction in false positives from 10 to 5, so the observed gain does not result from trading additional false alarms for recall in that split. Nevertheless, precision–recall trade-offs remain threshold-dependent and must be calibrated for practical warning systems. In safety-critical settings, missed detections may have serious consequences, but excessive false alarms can also reduce system usability. Larger and more diverse datasets, hard-negative mining, threshold calibration, and additional real-scene training are therefore needed before broader claims of real-world robustness can be made.

Beyond these evaluation-specific constraints, the study has several broader limitations. First, Synthetic-Debris currently covers only three debris categories—brick, paper box, and rock—and therefore represents only a subset of the objects that may occur on highways. Greater variation in object shape, material, scale, and appearance should be incorporated in future dataset construction. Second, although the synthetic images include different illumination periods, such as daytime, evening, and dusk, they do not adequately represent challenging conditions such as rain, fog, severe occlusion, strong reflections, or very long-range imaging. Third, despite its compact model size, FBENet exhibits higher wall-clock latency than YOLO11n, indicating that parameter count and FLOPs do not fully represent deployment speed. Its main advantage therefore lies in improved detection accuracy rather than faster inference, and further operator-level profiling and embedded-platform evaluation are needed.

Future work will expand the real road debris dataset with more object categories and a wider range of road environments, weather conditions, illumination levels, and camera viewpoints. Cross-scene, cross-device, and cross-dataset evaluation will be combined with domain adaptation and domain generalization methods to reduce the synthetic-to-real gap. Video-based temporal modeling and multimodal fusion using consecutive frames, infrared, depth, LiDAR, or millimeter-wave radar will also be investigated. In parallel, deployment-oriented evaluation will be conducted on representative embedded accelerators and complete perception pipelines, including latency, memory use, power consumption, thermal stability, and software-hardware compatibility.

## 6. Conclusions

(1) This study addresses highway debris detection under limited real data by combining dataset construction with a task-oriented redesign of YOLO11n. The Synthetic-Debris dataset contains three representative categories—brick, paper box, and rock—composited with diverse highway backgrounds. In FBENet, FreqFusion is placed at two top-down fusion stages, BiFPN performs weighted bidirectional aggregation, and EMA is applied only to the final high-resolution P3 feature. The contribution lies in the stage-specific organization of these operations for small, low-contrast debris rather than in proposing new primitive modules.
(2) On Synthetic-Debris, FBENet achieved 0.862 mAP@0.5, 0.664 mAP@0.5:0.95, and an F1-score of 0.811 with 2.43 M parameters and 6.8 GFLOPs. Ablation results show that FreqFusion-BiFPN mainly improves recall and small-target representation, whereas EMA shifts the precision–recall balance and yields the highest mAP@0.5 and mAP@0.5:0.95 but not the highest recall or F1-score. Runtime evaluation showed that FBENet was slower than YOLO11n under both PyTorch FP32 and TensorRT FP16, although it maintained relatively high GPU throughput after TensorRT optimization. Therefore, the proposed model should be understood as improving detection accuracy at the cost of additional latency, rather than improving inference speed. Its real-time suitability should be determined according to the target hardware and complete perception pipeline.
(3) Synthetic-to-real evaluation provides preliminary evidence of improved mean transfer performance under limited real samples. The fixed split yielded gains of 5.0 and 2.8 percentage points in mAP@0.5 and mAP@0.5:0.95, respectively, while five-fold cross-validation showed modest overall improvements and lower variance. Because the benefits varied by category and the real dataset contains only 110 images, broader claims about real-world robustness are not yet warranted. Further work should expand real-scene data and debris categories, reduce the synthetic-to-real domain gap, and evaluate the model on representative embedded hardware and complete highway-perception systems.

## Figures and Tables

**Table 1:** Comparison of tasks, parameters and calculations of YOLO11 series models [24].

Model	Task	Params/M	FLOPs/B
YOLO11n	Nano for small and lightweight tasks.	2.6	6.5
YOLO11s	Small model for higher accuracy while retaining efficiency.	9.4	21.5
YOLO11m	Medium model for general-purpose detection.	20.1	68.0
YOLO11L	Large model for higher accuracy at greater computational cost.	25.3	86.9
YOLO11x	Extra-large model for maximum accuracy and performance.	56.9	194.9

**Table 2:** Experimental environment and main hyperparameter settings.

Experimental Parameters	Value
Epoch	200
Batch size	8
Optimizer	SGD
Input image size	800
Initial learning rate	0.01
Momentum parameter	0.937
Weight decay	0.0005

**Table 3:** Module-level ablation results. P, R, and F1 denote precision, recall, and F1-score, respectively. Higher values of P, R, F1, mAP@0.5, and mAP@0.5:0.95 indicate better detection performance, whereas lower FLOPs and parameter counts indicate lower computational complexity. The best value in each column is highlighted in bold.

YOLO11n	FreqFusion-BiFPN	EMA	P	R	F1-Score	mAP@0.5	mAP@0.5:0.95	FLOPs(G)	Params/M
√			**0.851**	0.719	0.779	0.834	0.637	**6.3**	2.58
√	√		0.824	**0.803**	**0.813**	0.858	0.661	6.6	**2.41**
√		√	0.837	0.772	0.803	0.855	0.653	6.4	2.58
√	√	√	0.846	0.779	0.811	**0.862**	**0.664**	6.8	2.43

Note: A check mark (√) indicates that the corresponding component is included.

**Table 4:** Component-level ablation of the FreqFusion-BiFPN module on the Synthetic-Debris test set. A check mark (√) indicates that the corresponding component is included. The best result in each metric is shown in bold.

YOLO11n	FreqFusion	BiFPN	P	R	F1-Score	mAP@0.5	FLOPs(G)	Params/M
√			**0.851**	0.719	0.779	0.834	**6.3**	2.58
√	√		0.806	0.781	0.793	0.849	6.4	2.51
√		√	0.840	0.763	0.800	0.840	6.9	2.60
√	√	√	0.824	**0.803**	**0.813**	**0.858**	6.6	**2.41**

**Table 5:** Runtime performance of different model variants under PyTorch FP32.

Model	Preprocess (ms)	Inference (ms)	Postprocess (ms)	Mean E2E (ms)	P99 (ms)	FPS
YOLO11n	3.24	8.07	1.17	12.63	20.17	79.15
YOLO11n + FreqFusion-BiFPN	3.50	10.04	1.10	14.80	24.27	67.55
YOLO11n + EMA	4.05	9.27	1.20	14.70	27.27	68.01
FBENet	4.24	12.79	1.31	18.53	36.75	53.97

Note: Inference latency denotes network execution time only, whereas FPS is calculated using the total end-to-end wall-clock time, including preprocessing and postprocessing.

**Table 6:** Runtime performance of YOLO11n and FBENet under TensorRT FP16.

Model	Preprocess (ms)	Inference (ms)	Postprocess (ms)	Mean E2E (ms)	P99 (ms)	FPS
YOLO11n	3.24	2.65	1.23	7.27	10.25	137.64
FBENet	3.41	3.15	1.19	7.90	10.78	126.59

**Table 7:** Comparison of different attention mechanisms on the Synthetic-Debris dataset. The best result in each metric is shown in bold.

Model	mAP@0.5	mAP@0.5:0.95	FLOPs(G)	Params/M
YOLO11n	0.834	0.637	**6.3**	2.58
EMA-YOLO11n	**0.85** **5**	**0.653**	6.4	2.58
ECA-YOLO11n	0.841	0.636	6.3	2.58
SimAM-YOLO11n	0.826	0.630	6.3	2.58

**Table 8:** Ablation results for EMA placement. The best result in each metric is shown in bold.

Placement	Params/M	GFLOPs	P	R	mAP@0.5	mAP@0.5:0.95
None	2.58	6.3	**0.851**	0.719	0.834	0.637
P3	2.583	6.4	0.837	0.772	**0.855**	**0.653**
P4	2.583	6.4	0.816	0.757	0.834	0.630
P5	2.583	6.4	0.818	0.769	0.845	0.643
P3 + P4	2.584	6.4	0.801	**0.778**	0.834	0.635
P3 + P4 + P5	2.586	6.4	0.824	0.770	0.839	0.635

**Table 9:** Comparative results for different detection models. The best result in each metric is shown in bold.

Model	P	R	mAP@0.5	mAP@0.5:0.95	FLOPs(G)	Params/M	F1-Score
RT-DETR	0.717	0.643	0.665	0.441	103.4	32.0	0.678
YOLOv8n	0.766	0.768	0.825	0.625	8.1	3.0	0.767
YOLOv10n	0.789	0.778	0.829	0.625	8.2	2.7	0.783
YOLO11n	**0.85** **1**	0.719	0.834	0.637	6.3	2.6	0.779
YOLO12n	0.774	**0.794**	0.837	0.641	5.8	2.5	0.784
YOLOX [34]	0.551	0.638	0.582	-	36.8	7.25	0.591
ADWNet [35]	0.806	0.715	0.806	-	13.81	13.62	0.758
CIEFRNet [2]	0.796	0.771	0.828	0.636	33.2	14.1	0.788
RTMDet-tiny [36]	0.645	0.784	0.794	0.593	12.46	5.87	0.707
YOLO11-Dysample-P2	0.788	0.763	0.831	0.629	10.2	2.66	0.783
YOLO12-FreqFusionBiFPN-EMA	0.829	0.767	0.857	0.655	6.6	2.41	0.797
FBENet (ours)	0.846	0.779	**0.862**	**0.664**	6.8	2.43	**0.811**

**Table 10:** Synthetic-to-real transfer performance on the fixed real-scene split.

Model	mAP@0.5	mAP@0.5:0.95	Brick_AP	Paper Box_AP	Rock_AP
YOLO11n (Baseline)	0.598	0.259	0.577	0.962	0.254
FBENet (Ours)	0.648	0.287	0.660	0.995	0.290
Relative improvement	+8.36%	+10.8%	+14.4%	+3.4%	+14.2%

**Table 11:** Class-wise five-fold cross-validation results on the real-world dataset.

Model	Class	AP@0.5	VarAP@0.5	AP @0.5:0.95	VarAP@0.5:0.95	P	R
YOLO11n	brick	0.414 ± 0.129	0.0166	0.258 ± 0.101	0.0102	0.559 ± 0.294	0.333 ± 0.130
	paper box	0.477 ± 0.169	0.0284	0.249 ± 0.126	0.0158	0.626 ± 0.122	0.447 ± 0.193
	rock	0.346 ± 0.076	0.0058	0.130 ± 0.031	0.0010	0.510 ± 0.135	0.307 ± 0.081
	overall	0.412 ± 0.089	0.0080	0.213 ± 0.055	0.0030	0.565 ± 0.075	0.362 ± 0.092
FBENet (ours)	brick	0.465 ± 0.177	0.0312	0.291 ± 0.153	0.0234	0.707 ± 0.215	0.415 ± 0.164
	paper box	0.481 ± 0.159	0.0253	0.258 ± 0.121	0.0148	0.660 ± 0.265	0.423 ± 0.170
	rock	0.316 ± 0.051	0.0027	0.130 ± 0.028	0.0008	0.501 ± 0.132	0.313 ± 0.059
	overall	0.420 ± 0.077	0.0059	0.226 ± 0.049	0.0024	0.623 ± 0.176	0.383 ± 0.056

**Table 12:** Class-wise missed detections, false positives, and recall on the fixed real-scene validation split.

**Model**	**YOLO11n**	**FBENet**	**YOLO11n**	**FBENet**	**YOLO11n**	**FBENet**	**YOLO11n**	**FBENet**
Class	brick	brick	paper box	paper box	rock	rock	overall	overall
GT	8	8	5	5	51	51	64	64
TP	5	6	3	4	36	39	44	49
FN	3	2	2	1	15	12	20	15
Miss rate	37.5%	25.0%	40.0%	20.0%	29.4%	23.5%	31.3%	23.4%
R	62.5%	75.0%	60.0%	80.0%	70.6%	76.5%	68.8%	76.6%
FP	1	0	1	0	8	5	10	5
FP/GT	12.5%	0	20.0%	0	15.7%	9.8%	15.6%	7.8%
Precision	83.3%	100.0%	75.0%	100.0%	81.8%	88.6%	81.5%	90.7%

## Data Availability

The original data presented in the study will be openly available on GitHub at https://github.com/Chenyg0126/Synthetic-Debris.git (accessed on 16 July 2026) under the MIT License (code) and the CC BY-NC 4.0 License (data).
